# Brassinosteroids negatively regulate barley deacclimation tolerance via modulation of chloroplast gene expression and cell hydration

**DOI:** 10.1038/s41598-025-18844-8

**Published:** 2025-10-07

**Authors:** Ewa Pociecha, Magdalena Wójcik-Jagła, Agata Daszkowska-Golec, Michał Dziurka, Maciej T. Grzesiak, Damian Gruszka

**Affiliations:** 1 Department of Plant Breeding, Physiology and Seed Science, University of Agriculture in Kraków, Podłużna 3, 30-239 Kraków, Poland; 2 Institute of Biology, Biotechnology and Environmental Protection, University of Silesia in Katowice, Jagiellońska 28, 40-032 Katowice, Poland; 3 The Franciszek Górski Institute of Plant Physiology, Polish Academy of Sciences, Niezapominajek 21, 30-239 Kraków, Poland

**Keywords:** Plant sciences, Plant genetics, Plant physiology, Plant stress responses

## Abstract

Global warming may cause overwintering plants to lose tolerance to low temperatures owing to de-acclimation caused by winter temperature fluctuations. The effect of elevated temperatures is regulated by the endogenous levels of phytohormones, including brassinosteroids, which allow the maintenance of high productivity under stressful conditions This study examined barley (*Hordeum vulgare*) de-acclimation tolerance using two mutant lines: BW084, harbouring a mutation in the *HvCPD* gene, and BW312, harbouring a mutation in the *HvBRI1* gene. Both mutants were more tolerant to de-acclimation than the reference cultivar and displayed downregulation of chloroplast genes expression in response to de-acclimation, but differed in physiological traits related to photosynthesis and soluble carbohydrates metabolism. After 1 day of de-acclimation, biosynthesis mutant BW084 was characterized by a greater increase in the net photosynthesis rate than signalling mutant BW312. In turn after 10 days of de-acclimation, signalling mutant BW312 was characterized by the largest decrease in the fructan pool and degree of polymerization in leaves and crowns while biosynthesis mutant BW084 showed a significantly smaller decrease. In both mutants, de-acclimation tolerance was associated with maintenance of optimal tissue hydration, as evidenced by lower osmotic potential and higher cell hydration compared to the reference cultivar.

**Supplementary Information:**

The online version contains supplementary material available at 10.1038/s41598-025-18844-8.

## Introduction

In plants, many physiological and biochemical processes occur simultaneously and are regulated by internal and external factors. Environmental factors do not always occur at optimal levels; therefore, the survival of plants has developed many effective defence mechanisms^1^. It is particularly difficult to survive the winter period when low and sub-zero temperatures affect plants. The impact of low temperature of approximately 4–5 °C leading to an increase in frost resistance in plants, is known as cold acclimation. During cold acclimation, all supporting activities, mainly dehydration of the cells, increasement their osmotic potential, and increasing the concentration of carbohydrates, depend on temperature fluctuation and duration of these events^2^. De-acclimation, on the other hand, is defined as the process by which a plant loses the capacity to withstand subzero winter temperatures. Naturally, this process occurs when the plants prepare to leave the cold winter temperatures and begin to resume active growth as the daytime period lengthens and temperatures increase^3^. However, owing to climate change and warming periods, overwintering plants may experience de-acclimation during winter, before the beginning of spring. Thus, a temporary temperature rise during winter may lead to losses of winter hardiness previously attained through the cold acclimation process. De-acclimation may be reversible if re-exposure to low temperatures occurs. However, if exposure to warm temperatures persists long enough to result in a greater hydration of plant tissues and resumption of growth, an irreversible loss of frost resistance occurs^4^.

In addition to biochemical changes, low temperatures, slow down the growth and development process as reduced growth favours winter survival. Protecting a large number of leaves from frost is energy consuming, as photosynthesis is inhibited and the green parts are very sensitive to subzero temperatures^4^. Growth inhibition of the aboveground parts and induction of root system growth is also one of the reactions of plants to dehydration. In contrast, de-acclimation is associated with more intensive water uptake and growth intensification.

The effects of increased temperatures on the de-acclimation process are related to the endogenous levels of phytohormones. Brassinosteroids (BRs) are a group of hormones that enable plants to adjust to environmental changes such as temperature fluctuations, and allowing them to maintain high performance even under stress conditions. They may also play regulatory functions in the de-acclimation process^5^. The effects of BR also depend on their interference with other growth regulators, such as auxins and gibberellin.

In the light of the facts presented above, the aim of this study was to determine how mutations in genes affecting the signalling and synthesis of BRs influence the de-acclimation tolerance in barley. We verified the hypothesis that BRs affect photosynthesis and carbohydrate accumulation, as well as water relations and growth of barley plants in a way that modifies frost tolerance after de-acclimation. We also verified the hypothesis that temperature-dependent changes in the expression of differentially regulated genes contribute to tolerance to de-acclimation. This study also aimed to identify the mechanisms responsible for the differences between the mutants and their reference cultivars.

## Methods

In this study, we used two-rowed spring barley (*Hordeum vulgare *L.) cultivar Bowman (wild type) and two Near Isogenic lines (NILs) BW084 and BW312, which carry mutations in the BR biosynthesis, and signalling, genes respectively. The seeds were obtained from the collection of the Department of Plant Genetics and Functional Geomics, University of Silesia, Poland. The BW084 NIL (allele *brh13.p*) carries a missense mutation C2562T transition in the *HvCPD* gene which encodes C-23α-hydroxylase from the cytochrome P450 90A1 subfamily (CYP90A1) catalysing the early steps of BR biosynthesis. The mutation results in Pro-445 to Leu substitution, semi-dwarf phenotype of this NIL and reduced castasterone accumulation in BW084 compared to the Bowman cultivar^6^. In BW312 NIL (allele *ert-ii.79*), the mutant allele contains a double substitution CC1760/1761AA in the gene encoding BR receptor kinase, BRI1, which results in the Thr-573 to Lys substitution and is thought to prevent binding of BR molecules. As a result of this mutation (and the BRs insensitivity), BW312 accumulates more castasterone on the basis of the feedback mechanism, when compared to the Bowman cultivar^6^.

### Experimental design

Plants were sown in pots (30 cm wide and 15 cm long), and grown for 3 weeks in a greenhouse at 18 °C (day/night) in daylight (the experiment was conducted in November in Krakow, Poland, 50°03′N, 19°55′E). Next, the plants were placed in a growth chamber for cold acclimation at a temperature of 2/2 °C (day/night), with a photoperiod of 8/16 h (day/night) and 250 μmol m^−2^ s^−1^ PPFD for 3 weeks. After cold acclimation, plants were de-acclimated at 12/10 °C (day/night) 8/16 h (day/night) and 250 μmol m^−2^ s^−1^ PPFD. Light was provided by HPS Philips SON-T AGRO 400 W HPS lamps.

### Turgidity measurements

After collecting the fresh weight (FW) of the leaves, samples were immersed in distilled water in a closed container (100% RH) for 24 h. Subsequently, full turgor weight (FTW) was determined. Dry weight (DW) was the weight after 24 h of drying at 70 °C. Each measurement was performed in 6 replicates. Relative turgidity was calculated using the following formula: RT = [(FW–DW)/(FTW–DW)] × 100.

### Osmotic potential measurements

The osmotic potential of leaf cells was measured using an HR 33T Dewpoint Microvoltmeter (Wescor, Inc., USA) supplied with a C-52 Sample chamber. The cell sap was extracted with a fixed force from a leaf fragment (7 mm in diameter) collected from the youngest fully expanded leaf, and frozen in liquid nitrogen. Voltage measurements were performed after 20 min at dew point. The voltage is directly proportional to the water potential (for the given device, the proportionality factor is–0, 75 µV/bar, and 1 bar = 105 Pa). The measurement of the osmotic potential in the chamber was determined using the temperature coefficient of the thermocouples and calculated according to the formula πv_1_ = 0, 7(T_1_-T_0_) + πv_0_. Each measurement was performed in 6 replicates.

### Gas exchange measurements

Gas exchange parameters (P_N_: net photosynthesis rate; E: transpiration rate; g_S_: stomatal conductance) were measured using an IRGA analyzer (CIRAS-2, PP System, Amesbury, USA) with a Parkinson’s assimilation chamber (narrow leaf) with light attachment. An open system was used for the measurements. A flow rate of ambient air with a constant CO_2_ concentration (380 µmol mol^−1^) through the assimilation chamber amounted to 0.5 dm^3^ min^−1^. Chamber temperature was maintained below 25 °C until the photosynthesis rate stabilized. Photosynthetic capacity at light saturation was reached by exposing the leaves to PAR at 1000 µmol m^−2^ s^−1^. The intrinsic water use efficiency index (WUE) at the leaf level was calculated based on the ratio of CO_2_ assimilation and stomatal conductance values (P_N_/g_s_). Measurements were made from 11 a.m. to 1 p.m. Each measurement was performed in 6 replicates.

### Carbohydrate content and degree of polymerization analysis

The carbohydrates were analysed in accordance to the method reported by Jurczyk et al.^7^. Measurements for each treatment were performed on three independent pool samples, each consisting of leaves or crowns from six replicates. The collected plant material was preserved in liquid nitrogen, lyophilized in a freeze-dryer (LGA05, MLW, Leipzig, Germany; upgraded by JWE, Warsaw, Poland), and homogenized in a mixer mill (MM 400, Retsch, Haan, Germany). sample of 15 mg). The homogenized tissue was added to 1 ml of ultrapure water and heated for 5 min at 80 °C. The samples were then shaken for 15 min at 30 Hz (MM 400, Retsch) at ambient temperature for extraction. Next, the samples were centrifuged (15 min at 21,000×*g*), and the supernatant was subjected to the procedure described above. The supernatant was then divided into two portions. One was diluted with acetonitrile (1:1, v/v) and used for the determination of soluble sugars using high-performance liquid chromatography (HPLC), while the second one was used to estimate the fructan pool. The extracts were enzymatically hydrolysed using a mixture of exo-inulinase and endo-inulinase provided by Megazyme (Bray, Ireland). The carbohydrate extract (200 µL) was diluted with 200 mM sodium acetate buffer (1:1, v/v, pH 4.5), mixed with enzyme preparation (100 µL), and diluted with 100 mM sodium acetate buffer (1:5, v/v, pH 4.5). After overnight hydrolysis of the samples at 40 °C, the resulting suspension was diluted with acetonitrile (1:1, v/v), centrifuged, and analysed by HPLC. The formula of Verspreet et al.^8^ was used to calculate the mean degree of polymerization (DPav) of fructans: DPav = 1 + (Ff/Gf), where Ff and Gf are the molar concentrations of fructose and glucose released after the enzymatic cleavage of fructans. The interference of free glucose, fructose, sucrose, raffinose, and kestose was quantified by subtracting the glucose and fructose equivalents from the total pool of glucose and fructose measured after enzymatic hydrolysis. Free carbohydrates and fructooligosaccharide hydrolysates were analyzed via HPLC using an Agilent 1200 binary system (Agilent, Wolbrum, Germany) coupled to an electrochemical detector (Coulochem II, ESA, Chelmsford, MA, USA). Soluble carbohydrates (glucose, fructose, sucrose and other fructans) were separated on an RCX-10 column (7 µm, 250 mm × 4.1 mm; Hamilton, Reno, NV, USA) in a gradient mode of 75 mM NaOH solution and 500 mM sodium acetate in 75 mM NaOH solution, at a rate of 1.5 mL/min. Pulsed amperometric detection (analytical potential of 200 mV, oxidation potential of 700 mV, and reduction potential of-900 mV with respect to the palladium electrode) was applied to the gold electrode.

### RNA isolation

Leaves from each mutant and Bowman cultivar were sampled after cold acclimation (CA) and after 24 h after de-acclimation (DA) in three biological replicates (leaves from three different plants). The leaves were immediately frozen in liquid nitrogen after sampling and stored at − 80 °C until use. Each sample was collected from the middle part of the youngest fully developed leaf and weighted 0.03–0.05 g. Total RNA was isolated from 18 leaf samples using the RNeasy Plant Mini Kit (Qiagen, Hilden, Germany). The quantity and purity of the RNA were determined using a UV–Vis Q5009 spectrophotometer (Quawell, San Jose, CA, USA). RNA integrity was verified using a 2100 Bioanalyzer (Agilent Technologies, Santa Clara, CA, USA).

### RNA sequencing and differential expression analysis

Total RNA from 18 samples was submitted to Genomed (Warsaw, Poland) for sequencing. mRNA was isolated using oligo(dT)-attached magnetic beads, and cDNA libraries were constructed, converted into single-stranded circular DNA (ssCir DNA), and sequenced in paired-end 100 bp mode on the DNBSEQ platform (BGI, Cambridge, MA, USA), with sequencing depth of 20 mln pair reads (40 mln reads total). Raw reads were initially quality-checked with FastQC (Braham Bioinformatics, https://www.bioinformatics.babraham.ac.uk/projects/fastqc/), and then processed with Cutadapt^9^ to trim adapters and remove poor-quality sequences; quality control was re-assessed post-trimming. Cleaned paired-end reads were aligned to the BaRTv1 barley reference transcriptome^10^ using Kallisto version 0.43.0^11^, and expression was normalized to transcripts per million (TPM). Differential expression analysis comparing cold-acclimated and de-acclimated plants was performed with the Limma-Voom pipeline^12^, identifying genes with *p* < 0.01 and log_2_ fold changes > 1 or <  − 1 as differentially expressed. Dataset comparisons—between cold-acclimated BW084 versus BW, cold-acclimated BW312 versus BW, de-acclimated BW312 versus cold-acclimated BW312, and de-acclimated BW084 versus cold-acclimated BW084—were visualized using Venn diagrams (https://www.biotools.fr/misc/venny). Finally, differentially expressed genes were subjected to Gene Ontology analysis with ShinyGo (http://bioinformatics.sdstate.edu/go/) using an FDR ≤ 0.01, and results were visualized via bubble plots^13^.

### Testing of frost tolerance

Frost tolerance tests were carried out on de-acclimated plants of each genotype with four different frost treatments in darkness: − 8, − 10, − 11, and − 12 °C. Pots with plants were placed in a freezing chamber at a temperature of 2 °C. Then, the temperature was lowered by 3 °C/h until the required frost level was reached, and held at this level for a period of 6 h. Subsequently, the temperature was increased to 2 °C at the rate of 3 °C/h. After that, the pots with plants were transferred to a growth chamber and left for regrowth at 12 °C (8/16 h (day/night) photoperiod) and 150 μmol m^−2^ s^−1^ PPFD. After 3 weeks, plant survival was estimated using the visual score^14^. Estimation was done visually using a 0–9 point scale: 0 indicates completely dead plants with no signs of leaf elongation; 1—dying plants but with a leaf elongation of about 0.5 cm; 2—dying plants with a leaf elongation of about 1–2 cm; 3—dying plants with a leaf elongation of about 2 cm or more; 4—the plants may die or they may grow, but the inner leaves will be brown; 5—the plant may survive but the damage is visible, and the regrowing leaves will be discoloured and curled; 6—the plants survive, but damage is visible on about 50% of the leaves; 7—the plants are alive, but symptoms of freezing injuries are visible, and some of the leaves are discoloured or deformed; 8—only the tops of some of the inner leaves are discoloured or deformed, and 9—no symptoms of injury.

### Statistical analysis

The graphs were plotted using standard errors bars for each data point. Parametric data were analysed using multifunctional analysis of variance (ANOVA). A post-hoc comparison was conducted using Duncan’s multiple range test (*p* ≤ 0.05). Primary Component Analysis (PCA) was performed in ‘Multifactorial analysis’ module. Principal Component Analysis (PCA) was performed in ‘Multifactorial analysis’ module. All calculations were carried out using STATISTICA software (version 10.0; StatSoft, Inc., Tulsa, OK, USA).

## Results

### Physiological response of barley mutants in BR-related genes to cold de-acclimation

**Fig. 1:**
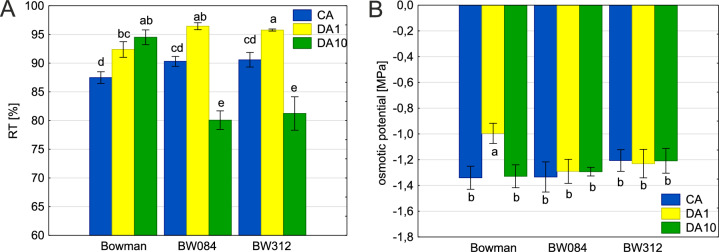
Relative turgidity (RT) (**A**) and osmotic potential (**B**) of cold acclimated (CA) and then de-acclimated for 1 day (DA1) and 10 days (DA10) plants. Significant differences between the cv. Bowman and NILs according to Duncan’s test, (*P* ≤ 0.05) are indicated by different letters.

To investigate the dynamics of tissue hydration during de-acclimation, we examined the response of cold-acclimated Bowman plants and Near-Isogenic Lines (NILs) with introgressed mutations in *HvBRI1* (BW312) and *HvCPD* (BW084) genes over time. After 1 day of de-acclimation, cold-acclimated (CA) Bowman plants exhibited significantly lower level of tissue hydration than NILs (Fig. 1A). Although 24 h of de-acclimation markedly increased water content in all genotypes, both NILs maintained higher hydration levels than Bowman, which eventually reached hydration levels comparable with those of the mutants after cold acclimation. Notably, after 10 d of de-acclimation, Bowman hydration continued to rise, whereas the mutants experienced a decline to levels below those noted for cold-acclimated plants.

In cold-acclimated (CA) plants, osmotic potential was similar between Bowman and both NILs (Fig. 1B). After one day of de-acclimation (DA1), Bowman exhibited an increase in osmotic potential, while the NILs remained unchanged. After 10 days of de-acclimation, Bowman’s osmotic potential returned to the its CA level, whereas NILs still showed no significant change.

**Fig. 2:**
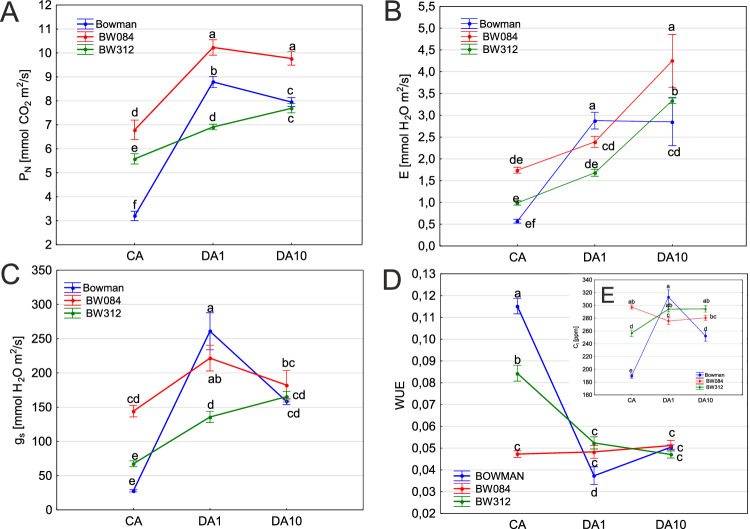
Net photosynthetic rate (mmol CO_2_ m^2^/s) (**A**), Transpiration (mmol H_2_O m^2^/s) (**B**), Stomatal conductance (mmol H_2_O m^2^/s) (**C**), WUE—photosynthetic water use efficiency (**D**) and intercellular CO_2_ concentration (ppm) (**E**), of previously cold acclimated and then de-acclimated plants. Significant differences between the cv. Bowman and NILs according to Duncan’s test, (*P* ≤ 0.05) are indicated by different letters.

After one day of de-acclimation (DA1), all cold-acclimated genotypes responded with an increased net photosynthesis (P_N_) (Fig. 2A). However, Bowman showed the largest increase, while BW312—impaired in BRs synthesis—showed the smallest. In contrast, BW084, with BR perception disruption, achieved the highest P_N_ overall. By 10 days (DA10), BW084 maintained its high P_N_, BW312 slightly increased, and Bowman’s P_N_ declined. At DA1, Bowman also showed the highest transpiration rate (E), stomatal conductance (g_S_), and intracellular CO_2_ concentration (C_i_) (Fig. 2B, C, E). Both NILs at this time did not significantly increase transpiration, but increased stomatal conductance. Notably, BW084 mesophyll CO_2_ decreased compared to pre-de-acclimation state, whereas in BW312 it increased (Fig. 2E). At DA10 similar levels of transpiration persisted in Bowman, whereas both g_S_ and C_i_ levels dropped. In contrast, both NILs increased transpiration and sustained their g_S_ and C_i_ levels. Following cold acclimation, Bowman exhibited the highest WUE, and BW084—the lowest (Fig. 2D). After one day of de-acclimation WUE decreased for all tested genotypes, yet the NILs displayed a higher value than Bowman, which later achieved similar levels after 10 days.

Alongside the differences in photosynthetic performance and water use efficiency, distinct changes in carbohydrate metabolism were also observed between genotypes tested. In CA plants, the oligofructans degree of polymerization in NILs was several times lower than in Bowman, with BW312 exhibiting the lowest values despite its high total fructan and oligofructan pool (Table 1). Bowman’s crowns contained the highest levels of complex sugars, such as raffinose and maltose, and those classified as fructans, sucrose (DP2), kestose (DP3), and nystose (DP4), and uniquely 111-5-kestose (DP5), likely driving its higher overall degree of polymerization of the total fructan pool. After 10 days of de-acclimation, simple sugar levels in the leaves dropped significantly in all genotypes, although Bowman maintained the highest levels of glucose, fructose, and sucrose and the largest overall fructan pool, with oligofructans reducing to zero. In Bowman nodes, the highest proportions of fructose (DP1), sucrose (DP2), and kestose (DP3) were found, along with the greatest total fructan polymerization, while the BW312 experienced the most pronounced reduction in fructan and DP pools.

**Table 1:** Carbohydrate content (nmol/mg) of cold acclimated (CA) and then de-acclimated (DA) leaves (L) and crowns (C) for 10 days barley plants. DP—average degree of polymerization Significant differences between the cv. Bowman and NILs according to Duncan’s test, (*P* ≤ 0.05) are indicated by different letters.

	nmol/mg		Inositol	Glycerol	Glucose	Fructose	Sucrose	Raffinose	Stachiose
	genotype					DP1	DP2		
CA	Bowman	L	nd	0.722 ef	80.367 f.	98.862 e	221.611 a	8.771 a	6.877 a
CA	BW084	L	0.011 e	0.780 ef	105.044 d	107.898 c	208.715 b	6.073 c	7.403 a
CA	BW312	L	0.571 a	3.403 d	110.502 c	139.751 a	177.589 c	7.515 b	4.467 b
CA	Bowman	C	0.027 e	5.947 b	149.999 b	107.757 c	119.714 f.	1.654 g	2.625 c
CA	BW084	C	0.485 b	7.829 a	156.282 a	115.791 b	72.786 h	1.090 h	2.673 c
CA	BW312	C	0.020 e	3.852 cd	112.051 c	36.423 g	45.670 j	2.057 f.	1.395 d
DA	Bowman	L	0.448 c	1.439 e	26.741 j	30.384 i	167.428 d	2.743 d	2.249 cd
DA	BW084	L	0.001 e	0.037 f.	17.952 k	13.430 k	135.637 e	2.095 f.	2.119 cd
DA	BW312	L	0.085 d	2.844 d	37.317 i	26.195 j	45.506 j	2.591 e	2.859 c
DA	Bowman	C	0.022 e	4.711 c	71.965 h	102.571 d	84.366 g	0.859 i	2.082 cd
DA	BW084	C	0.444 e	8.596 a	74.345 g	93.357 f.	37.277 k	0.980 hi	nd
DA	BW312	C	0.015 e	6.117 b	86.338 e	35.474 h	53.103 i	2.589 e	1.990 cd

	Kestose	Maltose	Nystose	111–5-kestose	Total fructans	Oligo-fructans	Oligofructans DP	Total fructans DP	
	DP3		DP4	DP5					
CA	2.804 e	9.906 a	2.657 e	nd	334.291 c	8.356 g	17.863 a	2.158 c	
CA	3.075 e	11.103 a	4.441 c	nd	364.656 b	40.527 d	4.315 bc	2.147 c	
CA	2.380 f.	11.153 a	2.850 de	nd	389.492 a	66.922 b	2.236 c	2.034 d	
CA	20.274 a	10.246 a	28.618 a	0.994 a	366.117 b	88.761 a	7.761 b	3.183 a	
CA	12.927 b	3.839 c	10.175 b	nd	291.364 d	79.685 a	4.059 bc	2.403 b	
CA	6.576 c	1.865 d	4.879 c	nd	149.284 i	55.736 bc	2.030 c	1.712 ef	
DA	1.031 h	7.363 b	0.003 g	0.703 b	215.439 f.	15.891 fg	0	1.723 ef	
DA	0.908 h	6.607 b	0.003 g	0.637 b	172.309 h	21.694 ef	0.327 c	1.702 ef	
DA	0.995 h	6.367 b	2.792 de	0.555 c	104.810 j	28.768 e	0	1.394 g	
DA	1.890 g	2.773 cd	1.722 f.	nd	238.302 e	47.754 cd	1.925 c	2.095 cd	
DA	2.440 f.	2.661 cd	1.853 f.	nd	198.971 g	64.044 b	0.984 c	1.752 e	
DA	4.347 d	2.849 cd	3.461 d	nd	180.230 h	83.846 a	1.513 c	1.626 f	

**Fig. 3:**
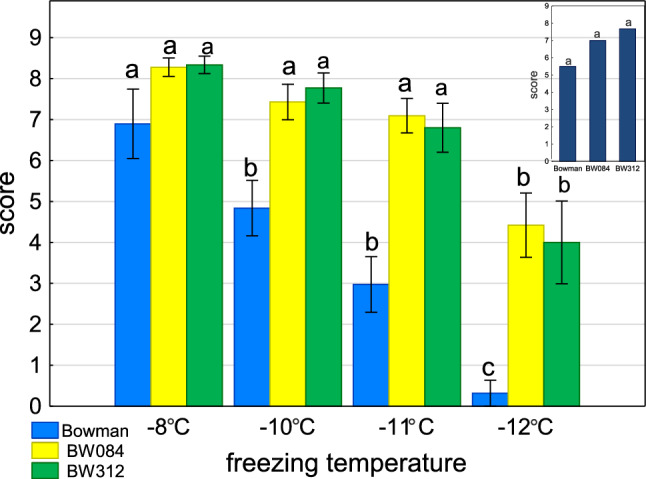
Score of regrowth after freezing test of previously cold acclimated and then de-acclimated for ten days plants. Significant differences between the cv. Bowman and its mutants according to Duncan’s test, (*P* ≤ 0.05) are indicated by different letters. In the insert, evaluation after one day of de-acclimation after freezing at − 10 °C.

The mean score of regrowth of the de-acclimated plants subjected to the freezing test decreased as the temperature decreased. The rate of decrease of the regrowth index value was much slower for both NILs than for Bowman. In Bowman a dramatically low value was reached at − 12 °C. For NILs at the same temperature, the regrowth index was significantly higher (around the mid-scale) (Fig. 3).

### Global transcriptomic reprogramming during barley cold de-acclimation

To investigate the transcriptomic responses to cold de-acclimation in barley, we analysed total RNA from leaves of cold acclimated (CA) and exposed to one day de-acclimation (DA) Bowman and NILs plants (Table 2). We identified a total of 507 significantly differentially expressed genes (DEGs) (282 upregulated and 225 downregulated) between BW084 and BW, and 1108 significantly differentially expressed genes (732 upregulated and 376 downregulated) between BW312 and BW in CA conditions. Notably, BW312 exhibited a greater magnitude of transcriptional reprogramming compared to BW084. Interestingly transferring CA barley NILs to warm conditions (DA) for 24 h led to increased number of DEGs, especially the down-regulated ones.

**Table 2:** Numbers of DEGs in comparisons between genotypes and between CA and DA. DEGs in comparisons of genotypes for 2 experimental variants (|log2(FC)|> 2, corrected P value < 0.05).

Genotype	Regulation	Treatment	
		CA	DA
BW084 v. BW	Up	282	469
	Down	225	1412
	Total	507	1881
BW312 v. BW	Up	732	1763
	Down	376	2453
	Total	1108	4216

**Fig. 4:**
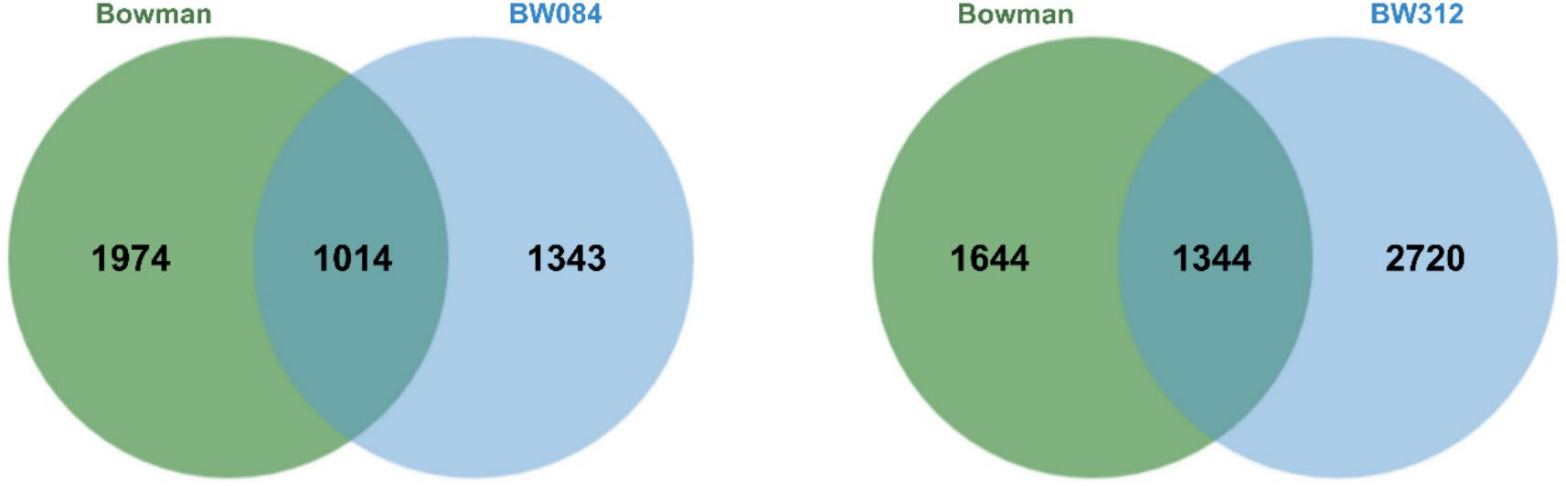
Venn diagram illustrating the number of differentially expressed genes that are common and distinct for each NIL (BW084, BW312, respectively) when compared to Bowman in de-acclimated conditions.

Both NILs, impaired BRs synthesis (BW084) and abnormal BRs perception (BW312) seem to undergo extensive global transcriptome changes after de-acclimation (CA versus DA state), with a pronounced trend toward gene expression downregulation when transitioning to warmer conditions (Fig. 4). After de-acclimation we identified 1014 DEGs that were common to BW084 and Bowman and 1344 DEGs that were common to BW312 and Bowman. A total of 1343 DEGs were specifically expressed in BW084 compared to Bowman, whereas 2720 genes were specific to BW312.

Among the DEGs common to Bowman and NILs after de-acclimation, BW312 was characterized by a distinctly higher proportion of genes regulated in the opposite direction than in Bowman (Fig. S1 and S2), which is in line with the much higher number of unique DEGs observed in this NIL (Fig. 4). Interestingly, genes regulated in a opposite direction to that of Bowman were also abundant in BW084 (Fig. S1).

Genes encoding transcription factors—such as WRKY (HORVU5Hr1G065380) (log_2_FC = 5.769 in BW084 and log_2_FC = 6.214 in BW312), cysteine protease (HORVU5Hr1G095580) (log_2_FC = 5.671 in BW084 and log_2_FC = 5.848 in BW312), molybdate transporter-like gene (HORVU7Hr1G078700) (log_2_FC = 5.496 in BW084 and log_2_FC = 6.82 in BW312), and stress response genes i.e. oxidoreductases HORVU6Hr1G009420 (log_2_FC = 5.711 in BW084 and log_2_FC = 5.621 in BW312), HORVU2Hr1G024280 (log_2_FC = 5.504 in BW084 and log_2_FC = 4.794 in BW312) were among the most upregulated genes in both NILs after one day of de-acclimation. In contrast, genes encoding chlorophyll-binding proteins e.g. HORVU6Hr1G016850 (log_2_FC = − 1.760 in BW084 and log_2_FC = − 3.176 in BW312), HORVU6Hr1G014500 (log_2_FC = − 1.417 in BW084 and log_2_FC = − 1.907 in BW312) and HORVU6Hr1G078480 (log_2_FC = − 1.293 in BW084 and log_2_FC = − 1.803 in BW312) in both NILs as well as HORVU7Hr1G058120 (log_2_FC = − 6,034), HORVU6Hr1G091650 (log_2_FC = − 6248) and HORVU6Hr1G091660 (log_2_FC = − 6,248)) in BW312, were among the most downregulated (Table S1-S4).

**Fig. 5:**
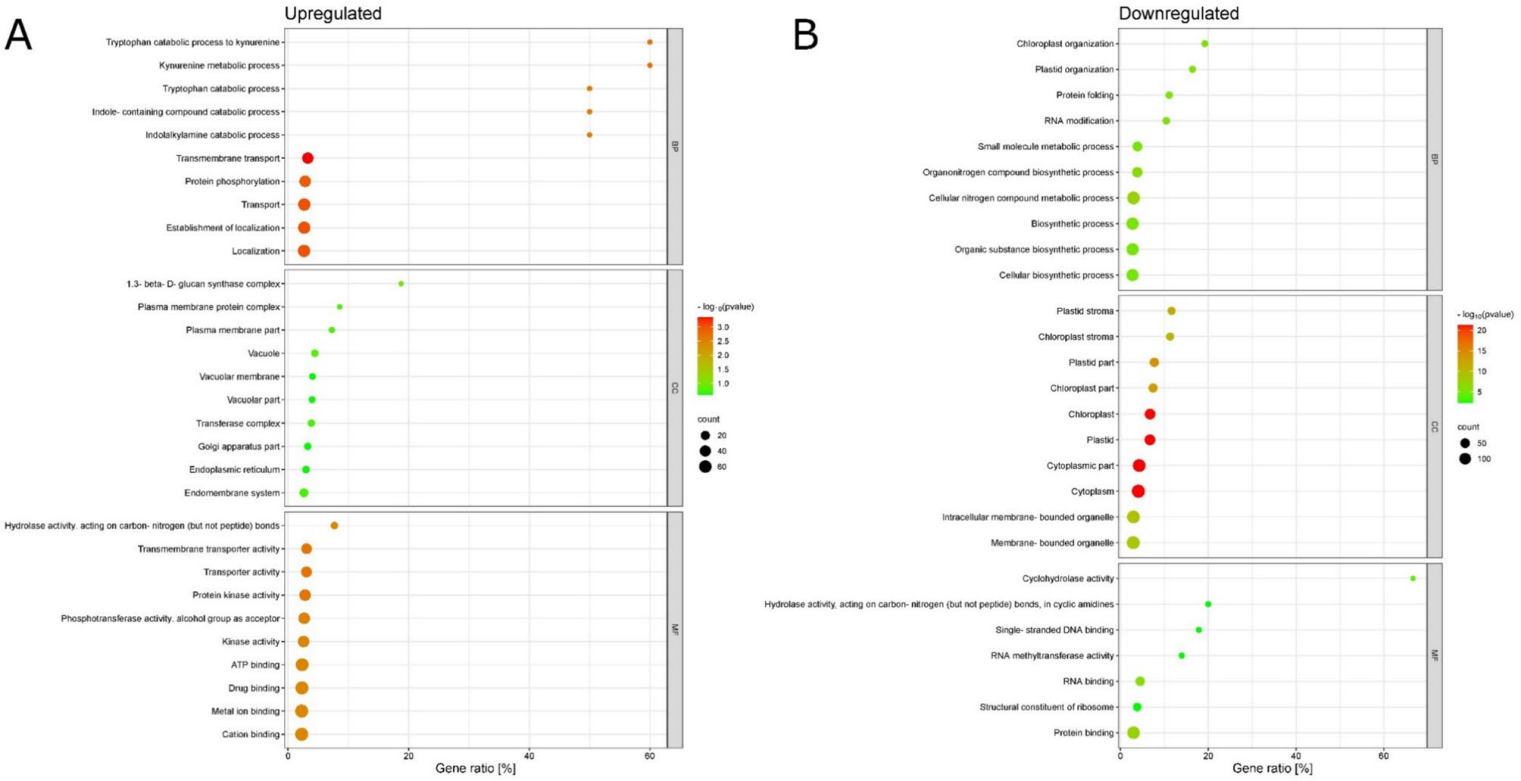
Transcriptomic gene ontology (GO) analysis of barley NIL BW084-specific (**A**) upregulated genes (**B**) downregulated genes after de-acclimation (FDR ≤ 0,01). Top 10 significantly enriched gene ontologies are reflected in this graph. Gene ontology terms are listed on the left whereas Gene Ratio (number of DEGs in the category related to the total number of genes in this category) is calculated and reflected on the x-axis. The size of the dots represents gene counts, and red symbolizes highly significant adjusted *P* value whereas green symbolizes the least significant *P* value.

**Fig. 6:**
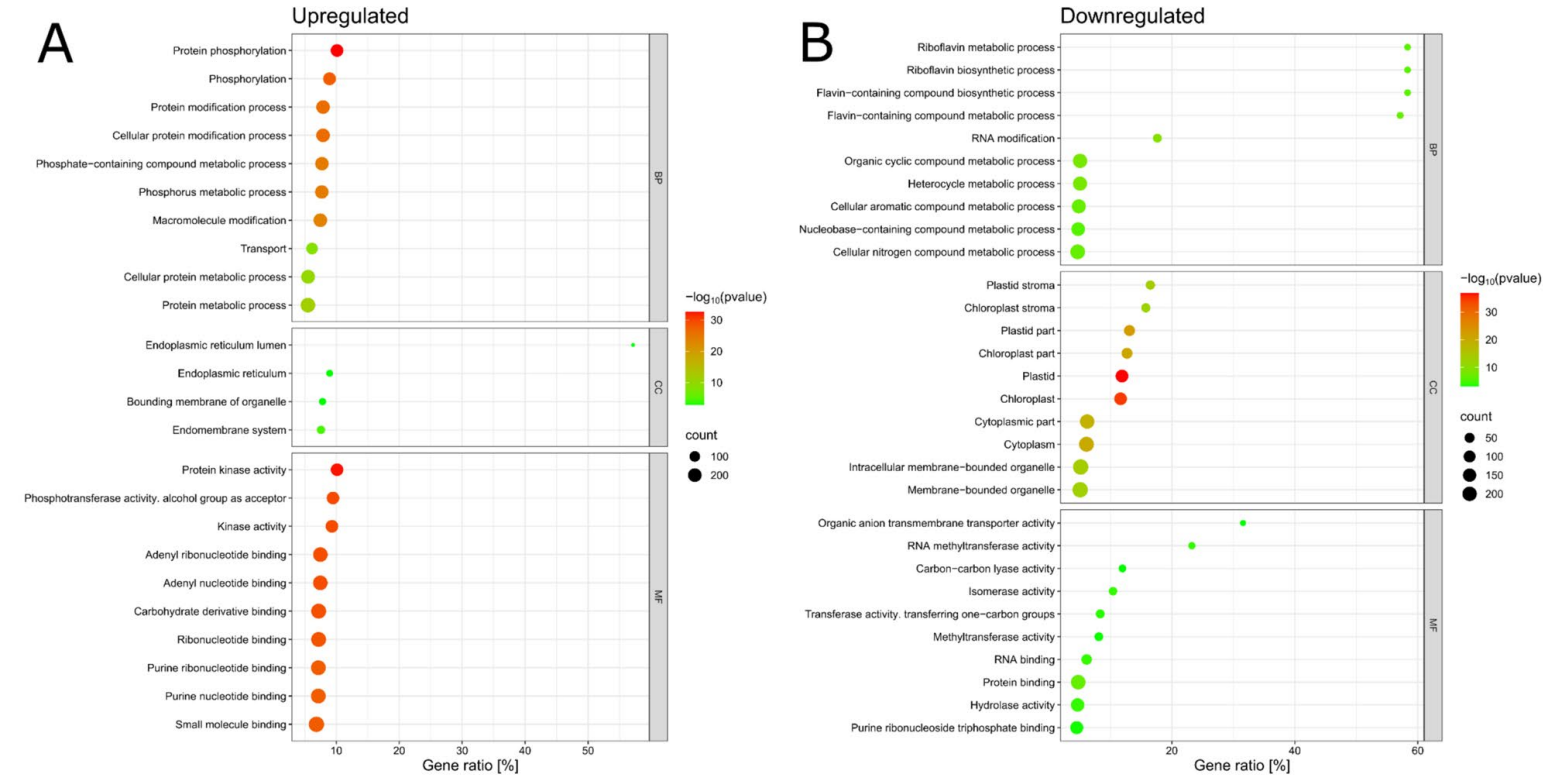
Transcriptomic gene ontology (GO) analysis of barley NIL BW312-specific (**A**) upregulated genes (**B**) downregulated genes after de-acclimation (FDR ≤ 0,01). Top 10 significantly enriched gene ontologies are reflected in this graph. Gene ontology terms are listed on the left whereas Gene Ratio (number of DEGs in the category related to the total number of genes in this category) is calculated and reflected on the x-axis. The size of the dots represents gene counts, and red symbolizes highly significant adjusted *P* value whereas green symbolizes the least significant *P* value.

We conducted Gene Ontology (GO) Enrichment analysis to understand the biological relevance of identified DEGs in BW084 and BW312, and their reference cultivar (Bowman). GO annotation analysis divided DEGs into biological process (BP), cellular component (CC), and molecular function (MF) categories (Figs. 5, 6). In BW084, the most significant GO categories overrepresented in the upregulated DEGs belonged to transmembrane transport, establishment of localization, and protein phosphorylation (BP), kinase activity, transmembrane transporter activity, ATP binding, hydrolase activity, and cation binding (MF), plasma membrane complex, endomembrane system, and vacuolar part (CC). GO terms for downregulated DEGs in BW084 included chloroplast organization, RNA modification, and protein folding (BP), RNA binding, and protein binding (MF), and cytoplasm, plastid, and chloroplast (CC) (Fig. 5). In the BW312 NIL the most significant GO terms for the upregulated DEGs were related to protein phosphorylation and modification, and transport (BP), protein kinase activity, small molecule binding, and nucleotide and ribonucleotide binding, including adenyl ribonucleotide binding (MF), and endoplasmic reticulum membrane (CC) (Fig. 6A). The downregulated gene group was most represented in the RNA modification, and riboflavin biosynthetic process (BP), RNA binding, and RNA methyltransferase (MF), as well as chloroplast, and plastid (CC) (Fig. 6B).

The most significantly upregulated genes specific to Bowman, in comparison to BW084, were related to plastids (chloroplast and plastid GO terms), whereas cellular components of chloroplasts (thylakoid membrane and photosystem I components) were the most abundant in terms of the total number of GO terms. Photosynthesis (light harvesting) and chlorophyll biosynthesis GO terms, as well as less abundant carbohydrate metabolic processes (biological process category) and chlorophyll binding, anion binding, hydrolase, and transferase activity (molecular function category), were less significant but highly abundant (Fig. S2A). In turn, the most significantly downregulated GO terms belonged to biological processes (phosphorylation, protein modification) and molecular function (ATP binding, carbohydrate derivative binding), whereas downregulated genes belonging to cellular components such as the Golgi apparatus, endoplasmic reticulum, nucleus, and membrane-bound organelle were less significant (Fig. S2B).

In the Bowman/BW312 comparison, the most significantly upregulated genes specific to Bowman in the biological process category were involved in carbohydrate metabolism (Fig. S2C). In the cellular component category, the number of DEGs in the chloroplast and plastid GO terms was the highest. In the molecular function category, the most abundant DEGs were annotated for hydrolase and transferase activities. The downregulated gene group was least represented by the cellular component category. In the biological processes category, protein and macromolecule modification and phosphorus metabolic process were the most significant GO terms, while in the molecular function category, the most significant GO terms were related to small molecule binding (such as anions, carbohydrate derivatives, nucleotides, etc.) (Fig. S2D).

**Fig.7:**
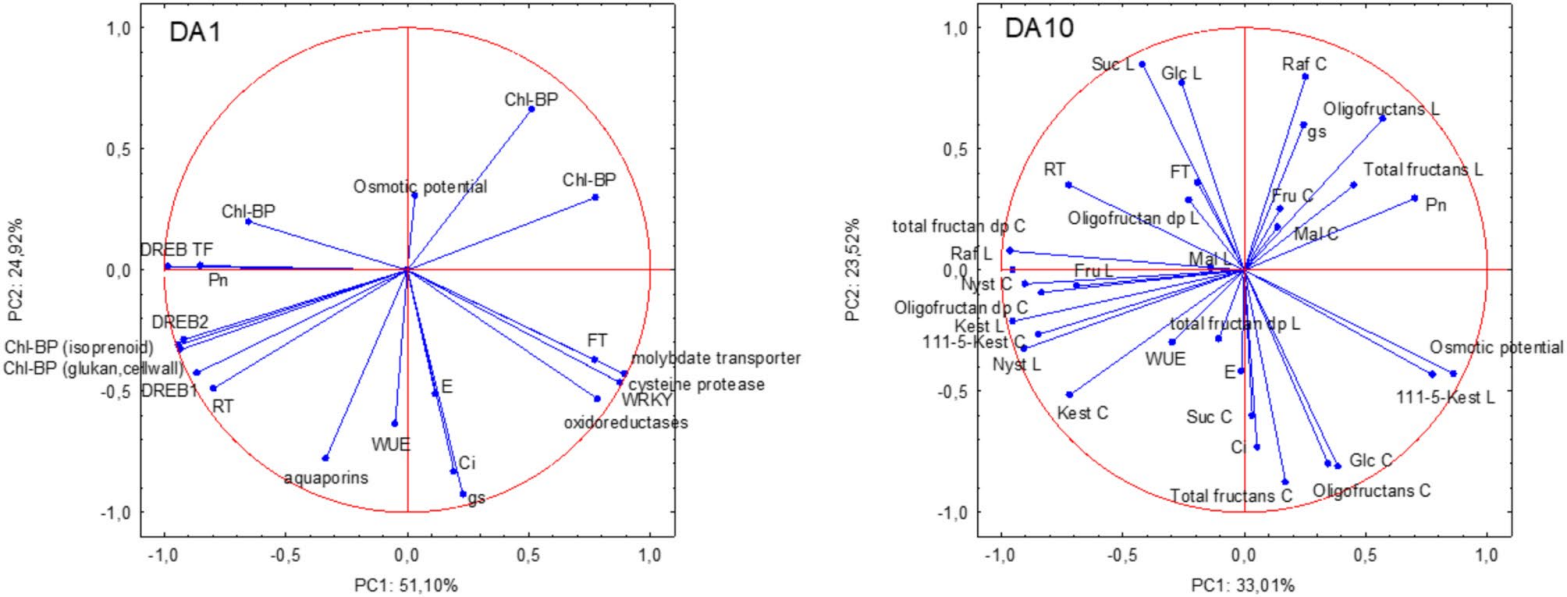
The first two principal components (PC1 and PC2) in plants de-acclimated for one (DA1) and ten days (DA10); sugar names: L—leaves, and C—crowns.

The first two principal components (PC1 and PC2) explained together 76.02% and 59.67% of the total variability in the plants de-acclimated for 1 and 10 days, respectively (Fig. 7). Expression of WRKY, cysteine protease, and molybdate transporter-like genes, and freezing tolerance (FT) displayed a positive correlation with PC1 after 1 day of de-acclimation while DREB1, DREB2, and some of the chlorophyll binding protein-coding genes (components of PSI, PSII and those involved in glucan biosynthesis) exhibited a negative correlation with PC1. Thus, higher tolerance to short one-day de-acclimation was accompanied by higher stomatal conductance, transpiration rate, osmotic potential, as well as higher expression of cysteine protease, WRKY and oxidoreductases genes. At the same time it was associated with lower hydration (RT) and net photosynthesis (Pn), lower expression of DREB1, DREB2, certain chlorophyll binding protein-coding and aquaporins genes. After 10 days of de-acclimation, physiological parameters such as osmotic potential, net photosynthetic rate (Pn), and stomatal conductance (gs) showed relatively strong positive correlations with PC1, while raffinose, kestose and nystose content in crowns exhibited negative correlations with this PC. Higher tolerance to prolonged ten-day de-acclimation showed a weak positive correlation with physiological parameters such as net photosynthetic rate (Pn), stomatal conductance (gs), and osmotic potential, as well as with the content of total fructans and oligofructans in leaves. Conversely, de-acclimation tolerance was weakly negatively correlated with the content of kestose, nystose, and raffinose in crowns.

A graphical summary of the most important results of the study is provided in Fig. S3.

## Discussion

Disturbances in both the synthesis and perception of brassinosteroids in NILs analysed in our study affected many parameters such as tissue hydration, photosynthesis, carbohydrate metabolism, and osmotic potential when compared to Bowman under cold acclimation and de-acclimation conditions. However, each NIL responded differently, indicating that different mechanisms are responsible for de-acclimation tolerance, which may be specifically dependent on the BRs biosynthesis or BRs signalling. Recognition of the nature of these changes may contribute to the knowledge of the mechanisms responsible for higher de-acclimation tolerance.

### De-acclimation tolerance—water status, carbohydrates and photosynthesis connections

De-acclimation occurring in mid-winter, driven mainly by temperature changes, is a key factor responsible for winter plant damage. De-acclimation is usually related to the start of rehydration and growth intensification, resulting in a reduction of freezing tolerance (Kosova et al. 2025)^15^. Studies on winter wheat and rye crowns revealed that higher hydration during de-acclimation was negatively correlated with frost resistance^15^. Growth cessation and cellular dehydration during winter protect plants from damage caused by unfavourable environmental factors as it reduces metabolic activity and energy consumption. In turn, the resumption of growth in spring occurs after the unfavourable environmental conditions have subsided and is associated with high hydration of cells.

Our previous experiments showed that deficiency of BRs biosynthesis and signalling genes resulted in reduced frost resistance after cold acclimation in BW084 and BW312 NILs^17^, while the same NILs after de-acclimation analyzed in the present study showed greater frost resistance than the reference cultivar Bowman. Interestingly BW084 and BW312 NILs cold-acclimated and exposed to frost (− 6 °C) and then the de-acclimated were also more frost resistant^18^. We suspect that this phenomenon is caused by differences in hydration resulting from BRs levels which have been shown to enhance drought tolerance and improve water use efficiency in plants. BRs can regulate plant hydration levels in several ways; via increasing the efficiency of water uptake by roots, e.g. through aquaporins^19^ or via regulation of water loss by stomata. In the model proposed by Xia et al.^20^ higher BRs concentrations increases the oxidised redox state and through interaction with abscisic acid induces stomatal closure, whereas lower concentrations and reduced glutathione redox state opens the stomata. Hydrophilic proteins and sugars are also responsible for maintaining water balance, the decrease of which is observed after the onset of de-acclimation^21^. The response to this decrease may be the increase in water channels (PIPs, tonoplastic V-ATPases) found after short-term de-acclimation (1–2 d) in the transcriptomic study on A. thaliana by Miki et al.^22^. Although aquaporins such as HORVU3Hr1G116790 (aquaporin gamma-TIP) and HORVU2Hr1G096360 (aquaporin PIP1-2 isoform X2) were up-regulated in all genotypes, the down-regulation of aquaporin HORVU7Hr1G081770 in both NILs may indicate a limiting tendency to increase hydration. On the other hand, it was found that there is a reallocation of resources towards plant growth, i.e. increased saccharide catabolism and lignin biosynthesis pathways in winter wheat treated with long-term de-acclimation^23^. Water management parameters appear to be important in assessing de-acclimation tolerance. The high correlation between dehydrin or relative protein transcripts accumulation (WCS120 protein family or WCS120 and WDHN13 transcripts in wheat and DHN5 protein in barley) and winter survival, can be used as a reliable marker of winter hardiness under field conditions at the beginning of winter, when plants have not yet finished vernalization^24^. Leaf relative turgidity (RT) is an important indicator of water status in plants. This reflects the current water content of the leaves in relation to the maximum water content they can hold at full turgor. In the present study, although it was only the first day of de-acclimation, all genotypes responded to an increase in temperature with an increase in hydration. In the following 10 days, the RT in the semi-dwarf mutants dropped to a level lower than that after cold acclimation, while in Bowman, RT remained high even with a tendency to rise. A decrease in hydration of the semi-dwarf mutants correlated with an increase in the transpiration rate and a decrease in WUE. Interestingly, BW084 and BW312 NILs showed delayed wilting compared to Bowman in response to drought stress^5^.

De-acclimation of plants during spring is associated with the cellular rehydration of plant tissue, which is accompanied by an increase in the metabolic rate and growth of cells. In contrast to spring, if hydration occurs in mid-winter due to temporary warming and then cold weather returns, this high-water content can result in mechanical damage caused by extracellular freezing. This can increase the rate of frost propagation through the tissues.

During de-acclimation, some of the metabolic regulation mechanisms, such as changes in the osmolyte concentration levels, are opposite to those during cold acclimation. The accumulation of osmoprotectant substances, such as soluble sugars and amino acids, which reduce the osmotic potential of cells, has been identified as an important factor in freezing tolerance. In Arabidopsis, after 24 h of de-acclimation, the rapid decrease in pool sizes of Glc, Fru, and Raf sugars proved to be functionally related to the rapid loss of freezing tolerance^25^. In the present study, the 1st day of de-acclimation caused an increase in osmotic potential, which occurred only in Bowman, while in NILs, osmotic potential remained at the same low level, probably due to the higher osmolyte content. The distinct carbohydrate profiles during de-acclimation appear to influence freezing tolerance. While Bowman maintained higher overall fructan levels and polymerization in its nodes, its regrowth index dropped dramatically at lower freezing temperatures (− 12 °C). In contrast, both NILs—with their lower oligofructan polymerization—experienced a much slower decline in regrowth index maintaining mid-scale values even at − 12 °C.

A decrease in temperature affects gene expression and protein activity related to photochemistry and CO_2_ fixation in overwintering green tissues. Cold acclimation is accompanied by the upregulation of photosynthesis-related genes, since higher activities of enzymes of the Calvin cycle and sucrose synthesis help avoid chilling-induced photodamage, which results from an overreduction of the photosynthetic electron transport chain^26,27^. Transcript levels of genes encoding chlorophyll a/b-binding protein, Rubisco subunits, ferredoxin-thioredoxin reductase, and transketolase genes were reported to be higher in cold-acclimated tissues than in non-acclimated tissues^28^. However, the increased resistance of cold-hardened leaves to chilling-induced photodamage is lost within three days of de-acclimation at higher temperatures^29^. In the present study, within the group of cold-acclimated plants, the lowest net photosynthesis rate was characteristic of Bowman and the highest of BW084, a mutant with disorders in brassinosteroid synthesis.

Photosynthesis does not seem to be a decisive factor for de-acclimation or re-acclimation because cold-acclimated winter wheat and barley lose the same amount of cold hardiness irrespective of de-acclimation occurring under light or dark conditions^30^. This observation was confirmed by the results of this study.

The elevated temperature that initiated the de-acclimation process simultaneously increased net photosynthesis, E, and Gs in all genotypes, which coincided with an increase in RT, reflecting the level of hydration. Stomata open with increasing temperature, leading to an increase in E, which usually decreases under low-temperature conditions because of the cold-related decrease in the water vapor pressure difference between the leaf surface and the atmosphere^31^. Although the photosynthetic apparatus response during de-acclimation was different in both NILs, BW084 showed the highest net photosynthetic rate and BW312 showed similar or lower net photosynthetic rate values than Bowman. Both NILs showed less damage in the freezing test than Bowman. After 10 d of de-acclimation, photosynthesis and Gs decreased only in Bowman. Although the decrease in photosynthesis is most often caused by water deficiency, mainly due to the limited access of plants to CO_2_ caused by the closing of stomata and the limitation of CO_2_ assimilation^3^, Bowman was the only one to increase RT, which decreased significantly in both NILs at the same time.

The indicator that combines the amount of carbon assimilated (P_N_) by plants and the amount of water used during this time (g_S_) is water use efficiency (WUE). At the leaf level, an increase in CO_2_ concentration leads to an increase in WUE until the leaf is exposed to temperatures that are not optimal for growth, then WUE begins a decrease^32^. In this study, Bowman was characterized by the highest WUE after cold acclimation, but after 1 day of de-acclimation, there was a sharp decrease to values ​​lower than those observed in both NILs, and after 10 days, there were no significant differences between genotypes. BW084 showed an interesting lack of reaction to temperature fluctuations; regardless of changes in temperature, it maintained WUE at the same level from cold acclimation until the 10th day of de-acclimation. This reaction seems to be beneficial since enhancing or at least not decreasing WUE may be one of the factors contributing to tolerance to de-acclimation resulting from winter warming. It was previously suggested that the reduced response to temperature rise might be responsible for the de-acclimation tolerance in barley^33^.

### De-acclimation tolerance and gene expression profile

During de-acclimation, plants quickly lose their freezing tolerance acquired during cold acclimation. Metabolite profiling of Arabidopsis Col-0 showed that the global metabolome composition after 24 h of de-acclimation approached that of the non-acclimated plants. Also, approximately half of the induced freezing tolerance is lost within 24 h after the return to 20 °C^34^. Therefore, our study focused on the transcriptome changes after the first 24 h of de-acclimation. Cold acclimated and then de-acclimated semi-dwarf barley NILs deficient in the BRs biosynthesis (BW084) and impaired BRs perception (BW312) showed significantly higher tolerance to de-acclimation and cellular desiccation than the Bowman cultivar. After one day of de-acclimation, the NILs transcriptomes exhibited more pronounced gene expression alterations—both upregulation and downregulation than in Bowman. Both the DEGs common to each of the NILs and Bowman and the genes specifically expressed only in each of the NILs hindered potential factors contributing to their higher tolerance to de-acclimation. However, metabolic pathways and transcript level changes in response to de-acclimation showed similarity in both NILs and at the same time great differences when compared to Bowman. Compared to Bowman, the results showed twice as many de-acclimation-related DEGs unique to BW312, and many more DEGs regulated in the opposite direction than in the case of BW084. This indicates that the mutation present in *HvBRI1* (BW312) affects more metabolic processes and pathways in response to de-acclimation than the *HvCPD* (BW084) mutation. It may result from the fact that the BR signalling is more intertwined through crosstalk hubs with other phytohormonal signalling pathways regulating plant response to environmental cues than the BR biosynthesis pathway^35–37^. Gene Ontology analysis of DEGs revealed similarities in response to de-acclimation, however, some differences between the NILs occurred as well.

In BW084, numerous genes controlling transmembrane transport, protein phosphorylation, transport, transmembrane transporter activity, protein kinase activity, ATP binding, and cation and metal ion binding were upregulated after one day of exposure to de-acclimation, which suggests that in BW084, more intensive transport across biological membranes might have occurred. In BW312, genes related to phosphorylation, protein modification, kinase activity, and ATP binding were upregulated, suggesting more efficient metabolic machinery. In both NILs, the most significantly downregulated genes were those encoding proteins localized in plastids and chloroplasts. This result was reflected in the net photosynthesis values, which increased to a lesser extent in NILs than in Bowman in response to elevated temperatures. Bowman, in comparison to both NILs, showed upregulation of chloroplast compounds, which was accompanied by the largest three-fold increase in net photosynthesis in response to one-day de-acclimation.

In Bowman (when compared to both NILs), downregulation of DEGs assigned to functions such as protein modification, protein phosphorylation, phosphorus metabolic process, carbohydrate derivative binding, nucleotide binding, and ATP binding may suggest its less efficient molecular machinery in terms of reaction to elevated temperature after one day of de-acclimation. Simultaneously, overrepresentation of the genes encoding hydrolase- and transferase-coding genes suggests temperature-induced hydrolysis reactions in Bowman.

After one day of de-acclimation, among the DEGs specific only to NILs (not found in Bowman), several showed substantial change in expression in relation to cold-acclimated plants. One of these DEGs was a specific gene encoding the transcription factor WRKY (HORVU5Hr1G065380), and it showed a high level of expression in both NILs. This gene was not identified as DEG in Bowman, although other genes from this large family of WRKY transcription factors, which create signalling webs that modulate many plant processes, were found to be downregulated. For example *Bh*WRKY1 transcription factors might be involved in regulating of the dehydration tolerance signalling pathway in the resurrection plant *Boea hygrometrica*^38^. On the other hand, WRKY46/54/70 cooperates with BES1, being one of the two major transcription factors regulating BR-dependent gene expression^39^ to regulate the expression of BRs target genes to promote growth. However it also negatively modulates drought tolerance by globally repressing drought-inducible gene expression^40^.

HORVU5Hr1G080300 (DREB1) and HORVU5Hr1G080330 (DREB2) were down-regulated in more tolerant to de-acclimation NILs in contrast to Bowman. Specific cold-induced signal transduction pathways interconnected with other dehydration stresses i.e. drought such as the DREB1 / CBF pathway, can be also modified by BRs. By inducing the transcription of drought response genes like CBF5 and dehydration-responsive element binding factors (DREBs), BRs enhance the drought tolerance of rapeseed seedlings (Gao et al. 2024)^41^.

The NILs were also highly abundant in senescence-specific cysteine protease-coding DEGs with possible functions of peptidase activity involved in senescence, programmed cell death, and response to fructose, sucrose, glucose, and phytohormones, such as cytokinins, ethylene, auxins, gibberellins, and abscisic acid. Among the most abundant DEGs in NILs, molybdate transporter-like genes (HORVU7Hr1G078700) points out to the possible role of molybdenum transport in de-acclimation tolerance. Molybdenum ions might be co-factors in enzymatic processes activated by the rise in temperature, and expression changes in genes related to molybdenum transport have been recently observed in de-acclimated wheat (Wójcik-Jagła et al. 2025, in preparation/data not published).

Genes involved in stress response, mostly peroxidase-coding genes were also amongst the most upregulated, specific to de-acclimated NILs, DEGs. Their high upregulation is in line with the hypothesis that de-acclimation is perceived as an opportunity to regenerate after stress, as shown by Wójcik-Jagła et al.^24^.

However, the lack of tolerance to de-acclimation in Bowman may be related to strongly enriched DEGs involved in cell growth and development processes. For example, the transcription factor GATA regulating cell differentiation, as well as the expansin and beta expansin genes, which play a key role in unidimensional cell growth, biosynthesis, and cell wall organization, were highly abundant in Bowman. The same expansin genes were strongly downregulated in BW312, but were not identified in BW084. Irreversible excessive cell growth induced by a temporary increase in temperature does not favor winterhardiness and may be the reason for lower de-acclimation tolerance.

Summarizing, both studied NILs demonstrated better cold de-acclimation response than Bowman. In response to de-acclimation, genes encoding proteins localized in chloroplasts or acting in photosynthesis process were downregulated in NILs, in contrast to the reference Bowman. Although in both NILs downregulated DEGs were enriched by plastid and chloroplast GO terms, their photosynthesis responses diverged. After one day of de-acclimation, BW084 exhibited higher net photosynthesis rate and a smaller reduction in the fructan pool and its degree of polymerization in leaves and nodes compared to BW312. In contrast, BW312 showed the lowest net photosynthesis and the most pronounced decline in fructan pool and degree of fructan polymerization, which probably resulted from the utilization of the reserves at lower photosynthetic efficiency. Furthermore, the enhanced de-acclimation tolerance in NILs was linked to their ability to maintain stage-dependent optimal tissue hydration levels, as evidenced by the reduced osmotic potential and relative turgidity (RT)—values that, after initially increasing after one day of de-acclimation, declined after 10 days to a level lower than those observed before cold acclimation. This means that brassinosteroids promote the cold acclimation process while at the same time reduce de-acclimation tolerance probably by enhancing ability to retain water, which can consequently intensify metabolism and stimulate growth processes. Since hydration-maintaining effect of BRs is temperature dependent—beneficial during cold acclimation (NILs with disturbances in BRs synthesis and perception show lower frost resistance) and unfavourable during de-acclimation (NILs with disturbances in BRs synthesis and perception show higher frost resistance), this phenomenon requires further research.

## Supplementary Information

Supplementary Information 1.

Supplementary Information 2.

Supplementary Information 3.

Supplementary Information 4.

Supplementary Information 5.

## Data Availability

The datasets generated and/or analysed during the current study are available from public databases. Raw data and TPM processed file are available from ArrayExpress (E-MTAB-1531) (https:/www.ebi.ac.uk/biostudies/arrayexpress), while the trimmed data (without adapters) are available from University of Agriculture in Krakow repository (10.15576/REPOURK/2025.1.2); link to download the research data: (https:/pliki.urk.edu.pl/index.php/s/jnsMc4ojIFDvSE9).
