# Supplementary material for: Brassinosteroids negatively regulate barley deacclimation tolerance via modulation of chloroplast gene expression and cell hydration
Source: Sci Rep. 2025 Oct 7;15:34971. doi: 10.1038/s41598-025-18844-8 (PMC12504425; doi:10.1038/s41598-025-18844-8)
Supplement: Supplementary file 1 — Supplementary Information 1. [file 41598_2025_18844_MOESM1_ESM.docx]

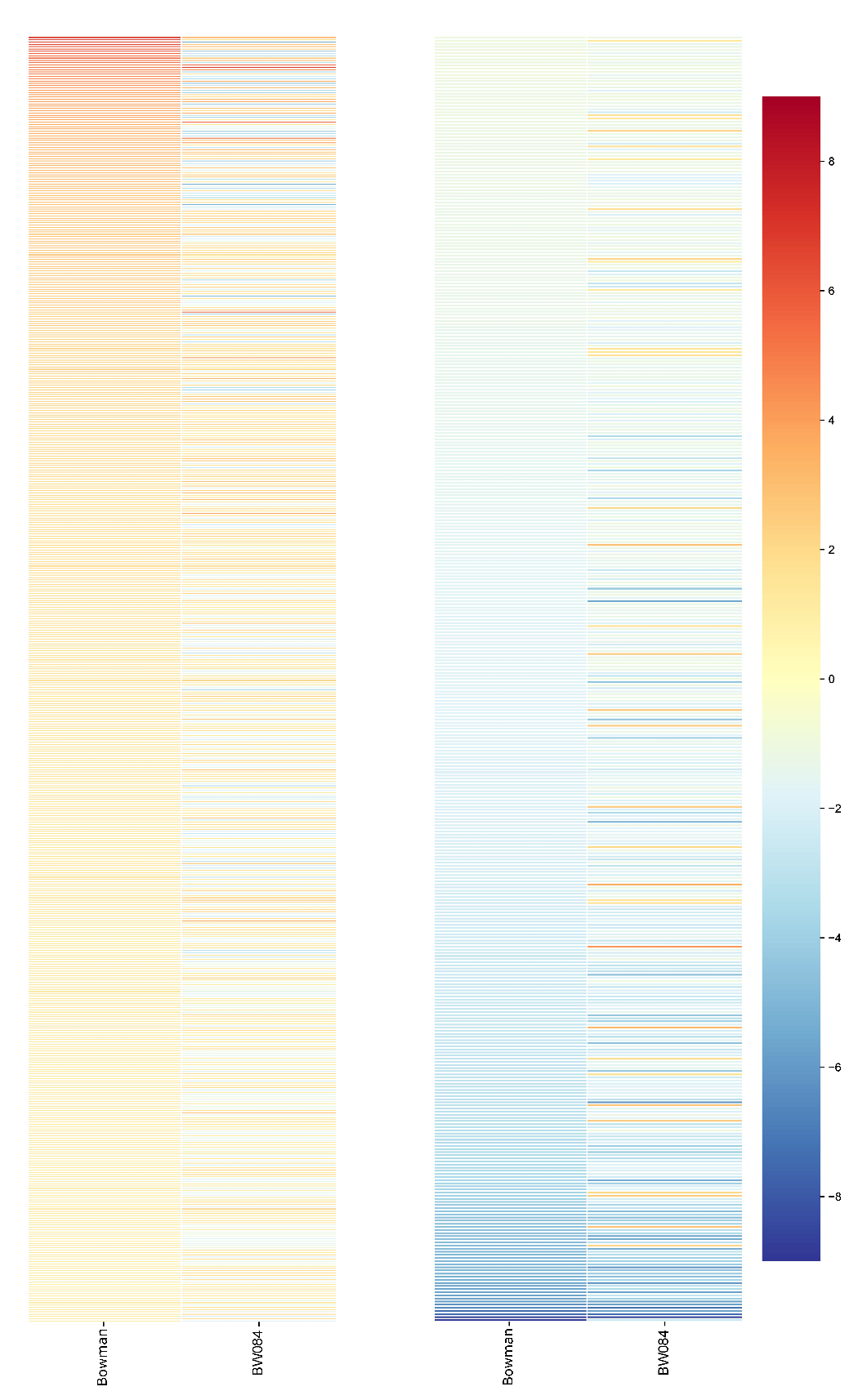


**Fig. S1.** Heatmap of differentially expressed genes (DEGs) between cold acclimated (CA) and de-acclimated (DA1) state in BW084 – barley NIL with mutation in the *HvCPD* gene compared to wild type plant – cultivar Bowman. Left – DEGs upregulated in Bowman, right – DEGs downregulated in Bowman.


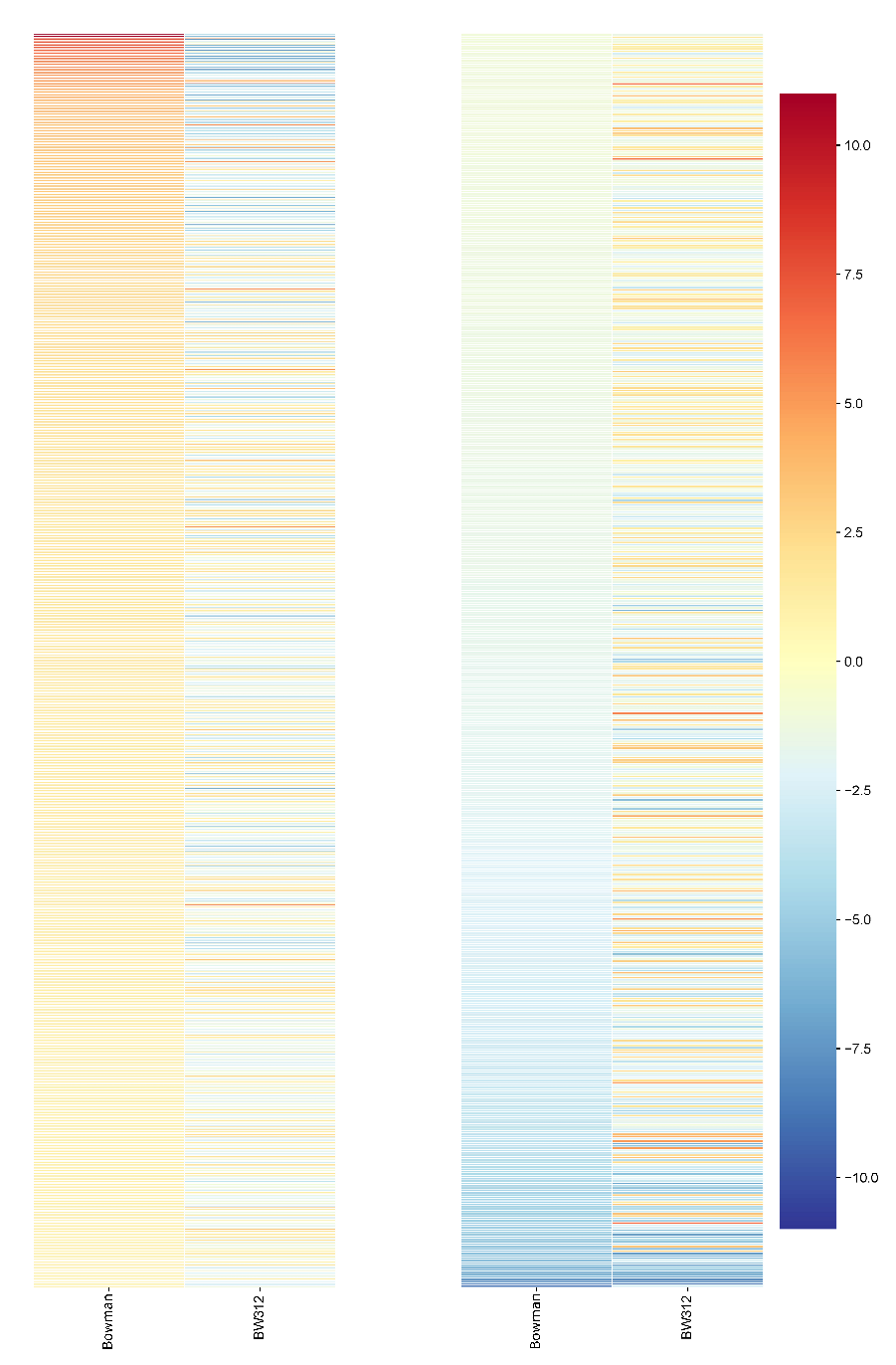


**Fig. S2.** Heatmap of differentially expressed genes (DEGs) between cold acclimated (CA) and de-acclimated (DA1) state in BW312 – barley NIL with mutation in the *HvBRI1* gene compared to wild type plant – cultivar Bowman. Left – DEGs upregulated in Bowman, right – DEGs downregulated in Bowman.
