# Supplementary material for: Brassinosteroids negatively regulate barley deacclimation tolerance via modulation of chloroplast gene expression and cell hydration
Source: Sci Rep. 2025 Oct 7;15:34971. doi: 10.1038/s41598-025-18844-8 (PMC12504425; doi:10.1038/s41598-025-18844-8)
Supplement: Supplementary file 2 — Supplementary Information 2. [file 41598_2025_18844_MOESM2_ESM.docx]

A B


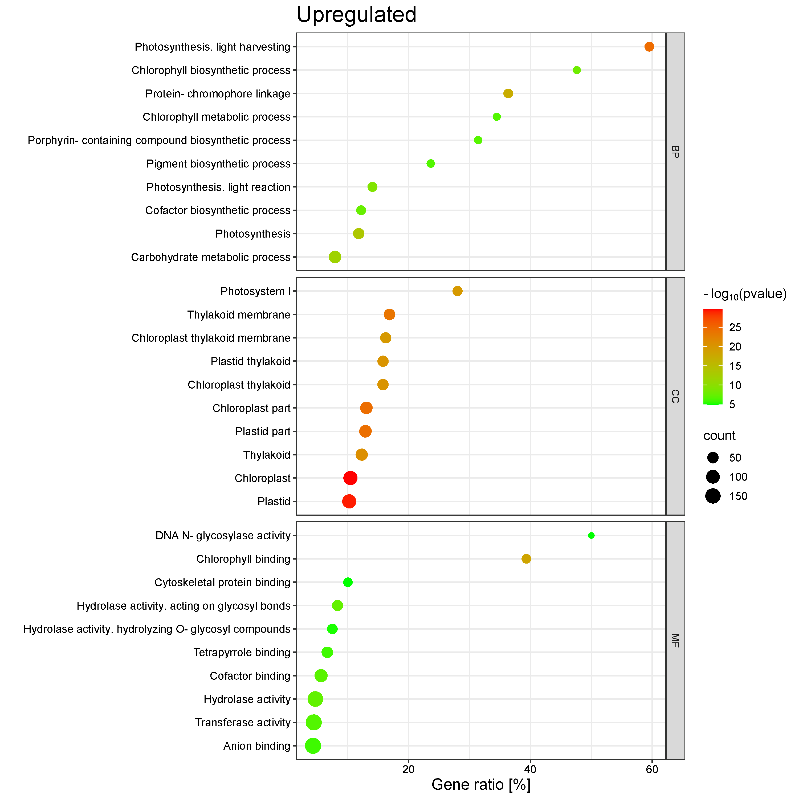

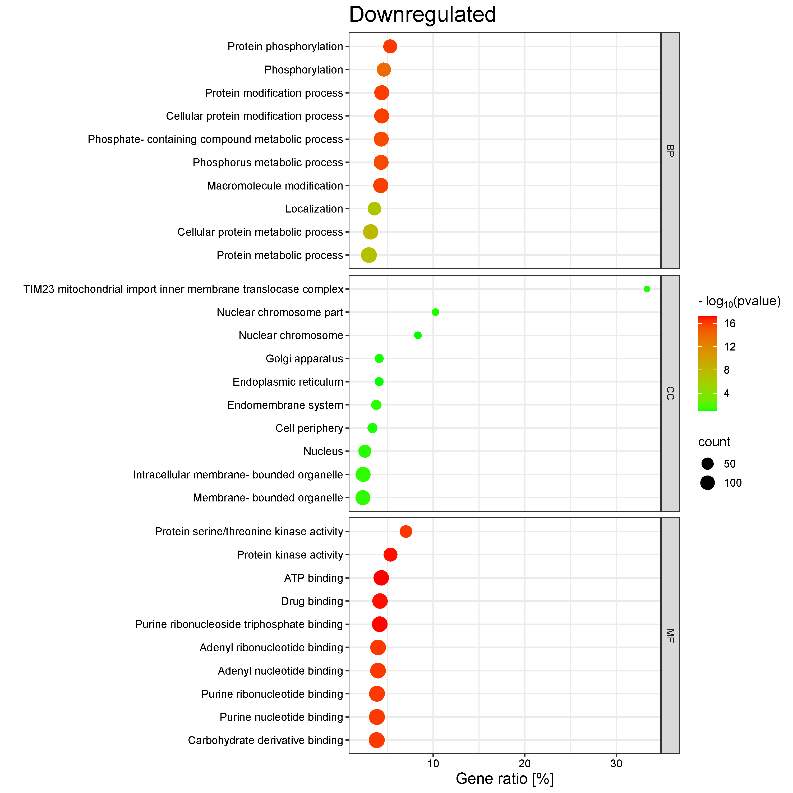


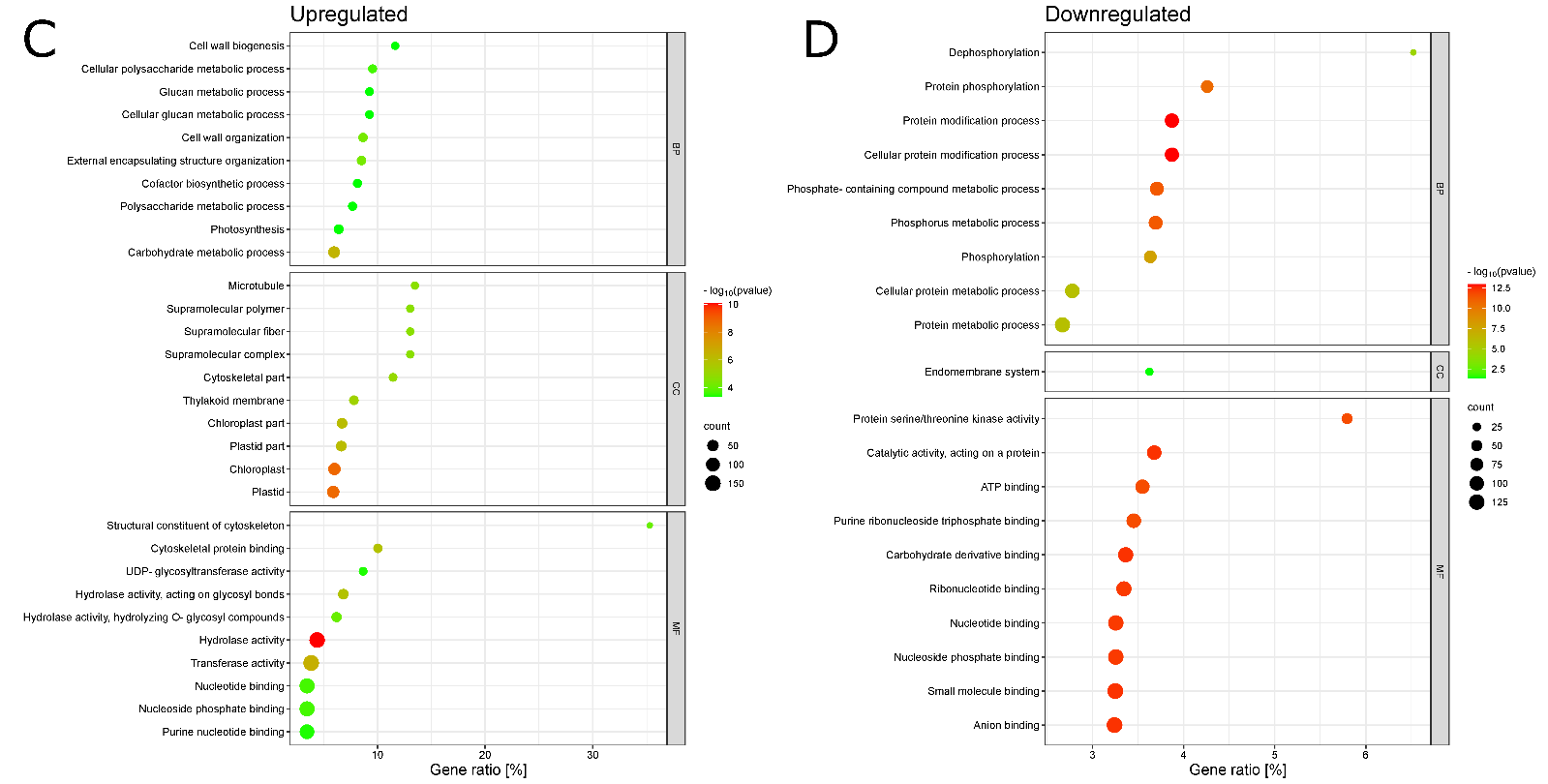


Figure S2. Transcriptomic gene ontologies (GO) analysis of barley Bowman cultivar after de-acclimation (A) in comparison to BW084 upregulated genes (B) in comparison to BW084 downregulated genes (C) in comparison to BW312 upregulated genes (D) in comparison to BW312 downregulated genes (FDR ≤ 0,01). Top 10 significantly enriched gene ontologies are shown in this graph. Gene ontology terms are listed on the left, whereas Gene Ratio (number of DEGs in the category related to the total number of genes in this category) was calculated and shown on the x-axis. The size of the dots represents gene counts, and red symbolizes highly significant adjusted *P* value, whereas green symbolizes the least significant *P* value.
