# Supplementary material for: Brassinosteroids negatively regulate barley deacclimation tolerance via modulation of chloroplast gene expression and cell hydration
Source: Sci Rep. 2025 Oct 7;15:34971. doi: 10.1038/s41598-025-18844-8 (PMC12504425; doi:10.1038/s41598-025-18844-8)
Supplement: Supplementary file 3 — Supplementary Information 3. [file 41598_2025_18844_MOESM3_ESM.docx]

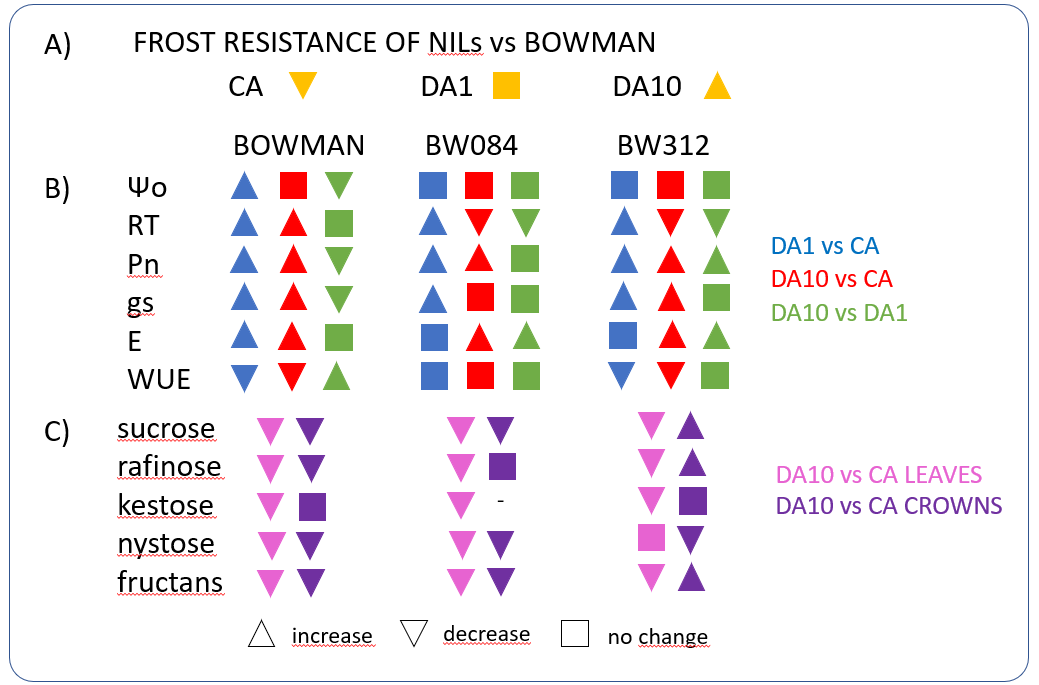


Figure S3. The main results of physiology analyses in Bowman and BW084 and BW312 NILs after cold acclimation (CA), one day de-acclimation (DA1) and ten days de-acclimation (DA10). (A) Frost resistance of both NILs BW084 and BW312 vs Bowman; (B) Water related and gas exchange parameters in comparison DA1 vs CA, DA10 vs CA and DA10 vs DA1 (C) main carbohydrates in comparison DA10 vs CA in leaves and crowns. Only statistically significant (p < 0.05) changes are presented.
