# Supplementary material for: Brassinosteroids negatively regulate barley deacclimation tolerance via modulation of chloroplast gene expression and cell hydration
Source: Sci Rep. 2025 Oct 7;15:34971. doi: 10.1038/s41598-025-18844-8 (PMC12504425; doi:10.1038/s41598-025-18844-8)
Supplement: Supplementary file 4 — Supplementary Information 4. [file 41598_2025_18844_MOESM4_ESM.docx]

| Table S1. Genes highly expressed in Bowman (log2FC  > 5 (-5) ) but undetectable in NILs (A); genes highly expressed at BW084 (log2FC  > 5 (-5) ) but undetectable in BW (B); genes highly expressed at BW312 (log2FC  > 5 (-5) ) but undetectable in BW (C); comparison of de-acclimated plants to cold acclimated plants.  **A.** Genes highly expressed in Bowman (log2FC  > 5 (-5) ) but undetectable in NILs (comparison of de-acclimated plants to cold acclimated plants) | | | |
| --- | --- | --- | --- |
| **Gene ID** | **log2FC** | **Annotation** | **GO terms** |
| HORVU7Hr1G010690 | 11,66218671 | Acid phosphatase 1 | RNA phosphodiester bond hydrolysis, endonucleolytic;nucleic acid binding;RNA-DNA hybrid ribonuclease activity;integral component of membrane;dephosphorylation;acid phosphatase activity; |
| HORVU1Hr1G088900 | 11,5048415 | Chlorophyll a-b binding protein, chloroplastic | metal ion binding;photosynthesis, light harvesting;photosystem II;integral component of membrane;chloroplast envelope;photosystem I;protein-chromophore linkage;chlorophyll binding;response to light stimulus;plastoglobule;chloroplast thylakoid membrane; |
| HORVU7Hr1G091490 | 10,85272141 | Small GTPase superfamily | Rab protein signal transduction;Golgi apparatus;cytoplasmic vesicle part;intracellular protein transport;cell plate;cell wall biogenesis;GTP binding;nucleus;ATP binding;endosome;GTPase activity;bounding membrane of organelle;integral component of membrane;whole membrane; |
| HORVU7Hr1G122470 | 10,73597729 | Sulfotransferase | sulfotransferase activity; |
| HORVU4Hr1G087310 | 8,69987965 | Acid phosphatase 1 | dephosphorylation;acid phosphatase activity; |
| HORVU6Hr1G084860 | 8,566914426 | Purple acid phosphatase | proton transmembrane transporter activity;metal ion binding;integral component of membrane;proton transmembrane transport;dephosphorylation;proton-transporting V-type ATPase, V1 domain;extracellular region;acid phosphatase activity; |
| HORVU3Hr1G086200 | 8,267563089 | Laccase | RNA phosphodiester bond hydrolysis, endonucleolytic;transmembrane transport;metal ion transmembrane transporter activity;nucleic acid binding;lignin catabolic process;RNA-DNA hybrid ribonuclease activity;copper ion binding;lignin biosynthetic process;apoplast;integral component of membrane;gene silencing by RNA;oxidation-reduction process;hydroquinone:oxygen oxidoreductase activity;metal ion transport; |
| HORVU7Hr1G089480 | 8,140423346 | Peroxidase | extracellular region;plant-type cell wall;hydrogen peroxide catabolic process;heme binding;peroxidase activity;response to oxidative stress;defense response to bacterium, incompatible interaction;plasmodesma;metal ion binding;cytoplasm;cellular oxidant detoxification;integral component of membrane;oxidation-reduction process; |
| HORVU7Hr1G040380 | 7,804231459 | Chlorophyll a-b binding protein, chloroplastic | nucleic acid binding;metal ion binding;photosynthesis, light harvesting;photosystem II;integral component of membrane;chloroplast envelope;photosystem I;protein-chromophore linkage;chlorophyll binding;response to light stimulus;plastoglobule;chloroplast thylakoid membrane; |
| HORVU7Hr1G040370 | 7,466023142 | Chlorophyll a-b binding protein, chloroplastic | photosynthesis, light harvesting;photosystem II;chloroplast envelope;photosystem I;regulation of transcription, DNA-templated;response to light stimulus;chloroplast thylakoid membrane;DNA binding;DNA-binding transcription factor activity;metal ion binding;integral component of membrane;protein-chromophore linkage;chlorophyll binding;plastoglobule; |
| HORVU2Hr1G000590 | 7,358408894 | Disease resistance protein RGA2 | ADP binding; |
| HORVU7Hr1G001030 | 7,335319782 | von Willebrand factor type A domain containing protein, expressed | 0 |
| HORVU6Hr1G091650 | 7,318967397 | Chlorophyll a-b binding protein, chloroplastic | photosynthesis, light harvesting;photosystem II;chloroplast envelope;photosystem I;regulation of transcription, DNA-templated;response to light stimulus;chloroplast thylakoid membrane;DNA binding;DNA-binding transcription factor activity;metal ion binding;integral component of membrane;protein-chromophore linkage;chlorophyll binding;plastoglobule; |
| HORVU6Hr1G091660 | 7,318967397 | Chlorophyll a-b binding protein, chloroplastic | photosynthesis, light harvesting;photosystem II;chloroplast envelope;photosystem I;regulation of transcription, DNA-templated;response to light stimulus;chloroplast thylakoid membrane;DNA binding;DNA-binding transcription factor activity;metal ion binding;integral component of membrane;protein-chromophore linkage;chlorophyll binding;plastoglobule; |
| HORVU3Hr1G087420 | 7,01743908 | Methyladenine glycosylase | nucleic acid binding;base-excision repair;integral component of membrane;glutamine metabolic process;methyltransferase activity;transferase activity;DNA-3-methylbase glycosylase activity;methylation; |
| HORVU4Hr1G074840 | 7,000128561 | Cytochrome P450 family protein, expressed | oxidoreductase activity, acting on paired donors, with incorporation or reduction of molecular oxygen;iron ion binding;sporopollenin biosynthetic process;integral component of membrane;oxidation-reduction process;monooxygenase activity;heme binding; |
| HORVU7Hr1G042010 | 6,977389839 | 3-ketoacyl-CoA synthase | endoplasmic reticulum membrane;nucleic acid binding;response to cold;zinc ion binding;unidimensional cell growth;transferase activity, transferring acyl groups other than amino-acyl groups;integral component of membrane;fatty acid biosynthetic process;response to light stimulus;wax biosynthetic process; |
| HORVU1Hr1G054200 | 6,874555305 | Beta-expansin 5 | cell wall;membrane;cell wall organization;sexual reproduction;extracellular region; |
| HORVU3Hr1G075210 | 6,825950938 | Expansin | unidimensional cell growth;structural constituent of ribosome;primary root development;cell wall;membrane;plant-type cell wall organization;extracellular region;ribosome;translation; |
| HORVU2Hr1G115980 | 6,821310604 | Lipoxygenase | chloroplast stroma;aldehyde-lyase activity;oxidoreductase activity, acting on single donors with incorporation of molecular oxygen, incorporation of two atoms of oxygen;long-chain fatty acid catabolic process;'de novo' IMP biosynthetic process;glutamine metabolic process;response to jasmonic acid;response to fungus;response to salicylic acid;response to ethylene;phosphoribosylformylglycinamidine synthase activity;mannosylglycoprotein endo-beta-mannosidase activity;carbohydrate metabolic process;response to abscisic acid;metal ion binding;oxylipin biosynthetic process;oxidation-reduction process; |
| HORVU0Hr1G026850 | 6,762398349 | 0 | mitochondrial gene expression;photosystem I;ribosome;intracellular organelle part;plastid;translation;RNA binding;structural constituent of ribosome;intracellular organelle lumen;mitochondrion;integral component of membrane;photosynthesis;transferase activity; |
| HORVU3Hr1G110330 | 6,751029698 | Phospholipase A1-Igamma1 chloroplastic | chloroplast;lipid metabolic process;organic substance catabolic process;hydrolase activity; |
| HORVU7Hr1G002820 | 6,74045301 | Heme peroxidase | oxidoreductase activity, acting on single donors with incorporation of molecular oxygen;response to oxidative stress;(R)-2-hydroxy-alpha-linolenic acid biosynthetic process;response to bronchodilator;systemic acquired resistance;cell death;NAD(P)H oxidase activity;cellular response to stress;monolayer-surrounded lipid storage body;cellular response to reactive nitrogen species;defense response to fungus;fatty acid catabolic process;transmembrane transport;transmembrane transporter activity;multi-organism cellular process;defense response to bacterium;heme binding;peroxidase activity;dioxygenase activity;prostaglandin-endoperoxide synthase activity;cellular response to inorganic substance;nucleus;response to abscisic acid;metal ion binding;oxylipin biosynthetic process;cellular oxidant detoxification;integral component of membrane;cellular response to salicylic acid stimulus;oxidation-reduction process;SCF complex assembly;lipid modification;protein ubiquitination; |
| HORVU1Hr1G024180 | 6,688094513 | Arabinogalactan protein 2 | somatic embryogenesis;response to salt stress;cell morphogenesis involved in differentiation;seed trichome differentiation;double-stranded DNA binding;regulation of transcription, DNA-templated;cell adhesion;plant-type secondary cell wall biogenesis;response to cytokinin;plasma membrane;response to auxin;response to gibberellin;unidimensional cell growth;response to abscisic acid;regulation of protein localization to cell surface;intrinsic component of membrane; |
| HORVU2Hr1G115960 | 6,660679243 | Lipoxygenase | chloroplast stroma;oxidoreductase activity, acting on single donors with incorporation of molecular oxygen, incorporation of two atoms of oxygen;long-chain fatty acid catabolic process;jasmonic acid biosynthetic process;'de novo' IMP biosynthetic process;glutamine metabolic process;response to wounding;response to jasmonic acid;plastid stroma;response to salicylic acid;phosphoribosylformylglycinamidine synthase activity;response to cold;chloroplast;plastid thylakoid;lateral root formation;green leaf volatile biosynthetic process;aldehyde-lyase activity;RNA phosphodiester bond hydrolysis, endonucleolytic;catalase activity;negative regulation of defense response to insect;response to fungus;circadian rhythm;response to ethylene;plasma membrane;heme binding;nucleic acid binding;RNA-DNA hybrid ribonuclease activity;mannosylglycoprotein endo-beta-mannosidase activity;carbohydrate metabolic process;response to abscisic acid;metal ion binding;oxylipin biosynthetic process;integral component of membrane;cellular oxidant detoxification;oxidation-reduction process;lipid modification; |
| HORVU5Hr1G000980 | 6,490384924 | Leucine-rich repeat protein kinase family protein isoform 1 | oxidoreductase activity, acting on paired donors, with incorporation or reduction of molecular oxygen;protein serine/threonine kinase activity;protein phosphorylation;plasma membrane;heme binding;ATP binding;activation of MAPKK activity;protein binding;transmembrane receptor protein tyrosine kinase activity;iron ion binding;integral component of membrane;cellulose biosynthetic process;oxidation-reduction process;peptidyl-tyrosine modification;intracellular signal transduction; |
| HORVU1Hr1G039250 | 6,433014499 | Cellulose synthase | seed trichome differentiation;UDP-glucose metabolic process;translational initiation;plant-type secondary cell wall biogenesis;plasma membrane;plant-type primary cell wall biogenesis;plant-type cell wall biogenesis;translation initiation factor activity;DNA binding;nucleus;trans-Golgi network;metal ion binding;mitotic cytokinesis;integral component of membrane;cellulose synthase (UDP-forming) activity;cellulose biosynthetic process;cell wall organization;ligase activity; |
| HORVU4Hr1G081040 | 6,403033727 | Pleiotropic drug resistance protein 2 | ATPase activity;ATP binding;ATPase-coupled transmembrane transporter activity;transmembrane transport;integral component of membrane;plasma membrane; |
| HORVU6Hr1G024860 | 6,37594525 | Tyrosine-sulfated glycopeptide receptor 1 | ATP binding;integral component of membrane;protein serine/threonine kinase activity;protein phosphorylation; |
| HORVU2Hr1G010690 | 6,352082699 | Ribulose bisphosphate carboxylase small chain | carbohydrate biosynthetic process;photorespiration;photosynthesis;oxidation-reduction process;carbon fixation;monooxygenase activity;ribulose-bisphosphate carboxylase activity;plastid; |
| HORVU2Hr1G010690 | 6,341213856 | Ribulose bisphosphate carboxylase small chain | carbohydrate biosynthetic process;photorespiration;photosynthesis;oxidation-reduction process;carbon fixation;monooxygenase activity;ribulose-bisphosphate carboxylase activity;plastid; |
| HORVU3Hr1G081180 | 6,295619721 | Expansin | unidimensional cell growth;structural constituent of ribosome;primary root development;cell wall;membrane;plant-type cell wall organization;extracellular region;ribosome;translation; |
| HORVU0Hr1G005360 | 6,262984591 | Carboxypeptidase | secondary metabolic process;proteolysis;serine-type carboxypeptidase activity; |
| HORVU3Hr1G034100 | 6,24365124 | Germin-like protein 5-1 | protein serine/threonine phosphatase activity;regulation of root development;plasmodesma;oxidoreductase activity;apoplast;cell wall;protein dephosphorylation;integral component of membrane;manganese ion binding;oxidation-reduction process;nutrient reservoir activity;plasmodesmata-mediated intercellular transport; |
| HORVU2Hr1G010690 | 6,201461391 | Ribulose bisphosphate carboxylase small chain | DNA-dependent DNA replication;photorespiration;monooxygenase activity;plastid;DNA binding;ATP binding;DNA-directed DNA polymerase activity;carbohydrate biosynthetic process;DNA biosynthetic process;photosynthesis;oxidation-reduction process;carbon fixation;ribulose-bisphosphate carboxylase activity; |
| HORVU3Hr1G027970 | 6,158194437 | Tyrosine--tRNA ligase | plasmodesma;integral component of membrane;Golgi apparatus;vacuole;ligase activity;O-acetyltransferase activity; |
| HORVU2Hr1G079910 | 6,117204594 | Chlorophyll a-b binding protein, chloroplastic | protein domain specific binding;photosynthesis, light harvesting;photosystem II;chloroplast envelope;photosystem I;response to light stimulus;chloroplast thylakoid membrane;oxidoreductase activity;integral component of membrane;protein-chromophore linkage;oxidation-reduction process;chlorophyll binding;plastoglobule; |
| HORVU2Hr1G013740 | 6,079144366 | Peroxidase | chromosome segregation;protein kinase activity;microtubule cytoskeleton organization;extracellular region;plant-type cell wall;protein phosphorylation;mitotic cell cycle;hydrogen peroxide catabolic process;heme binding;peroxidase activity;response to oxidative stress;ATP binding;plasmodesma;rhythmic process;metal ion binding;cellular oxidant detoxification;integral component of membrane;regulation of cytokinesis;cytosol;oxidation-reduction process;defense response to fungus; |
| HORVU2Hr1G090670 | 6,069585135 | Sulfotransferase | nucleic acid binding;zinc ion binding;integral component of membrane;sulfotransferase activity;hydrolase activity; |
| HORVU1Hr1G054240 | 5,989326465 | Beta-expansin 7 | cell wall;membrane;cell wall organization;sexual reproduction;extracellular region; |
| HORVU4Hr1G056220 | 5,930925054 | Protein trichome birefringence-like 28 | histone H4 acetylation;response to salt stress;Golgi apparatus;intracellular organelle part;chromatin silencing at telomere;response to water deprivation;xylan biosynthetic process;chromosome, telomeric region;nuclear chromatin;positive regulation of transcription, DNA-templated;histone binding;bounding membrane of organelle;integral component of membrane;response to freezing;H4 histone acetyltransferase activity;O-acetyltransferase activity; |
| HORVU4Hr1G090820 | 5,918503753 | Xyloglucan endotransglucosylase/hydrolase | cell wall macromolecule catabolic process;metal ion binding;apoplast;cell wall;integral component of membrane;cell wall organization;xyloglucan:xyloglucosyl transferase activity;hydrolase activity, hydrolyzing O-glycosyl compounds;carbohydrate binding;xyloglucan metabolic process;plasma membrane;cell wall biogenesis; |
| HORVU1Hr1G088920 | 5,870075514 | Chlorophyll a-b binding protein, chloroplastic | photosynthesis, light harvesting;photosystem II;chloroplast envelope;photosystem I;regulation of transcription, DNA-templated;response to light stimulus;chloroplast thylakoid membrane;nucleic acid binding;DNA binding;DNA-binding transcription factor activity;metal ion binding;integral component of membrane;protein-chromophore linkage;chlorophyll binding;plastoglobule; |
| HORVU5Hr1G001180 | 5,867440523 | Lipoxygenase | oxidoreductase activity, acting on single donors with incorporation of molecular oxygen, incorporation of two atoms of oxygen;jasmonic acid biosynthetic process;response to wounding;response to jasmonic acid;plastid stroma;response to fungus;response to herbivore;response to salicylic acid;circadian rhythm;plastid envelope;response to ethylene;response to cold;chloroplast;response to bacterium;protein binding;response to abscisic acid;metal ion binding;oxylipin biosynthetic process;positive regulation of gene expression;plastid thylakoid membrane;integral component of membrane;oxidation-reduction process;green leaf volatile biosynthetic process;lipid modification; |
| HORVU1Hr1G078740 | 5,860334973 | 1-aminocyclopropane-1-carboxylate oxidase 3 | oxidoreductase activity;metal ion binding;oxidation-reduction process; |
| HORVU4Hr1G001450 | 5,8295488 | Fatty acyl-CoA reductase | alcohol-forming fatty acyl-CoA reductase activity;lipid metabolic process;integral component of membrane;oxidation-reduction process;fatty-acyl-CoA reductase (alcohol-forming) activity;suberin biosynthetic process;long-chain fatty-acyl-CoA metabolic process; |
| HORVU1Hr1G089380 | 5,827449801 | Subtilisin-like protease | extracellular space;proteolysis;serine-type endopeptidase activity;integral component of membrane; |
| HORVU5Hr1G093410 | 5,795188564 | Leucine Rich Repeat family protein, expressed | integral component of membrane;ADP binding;phosphorylation;structural constituent of cell wall;kinase activity; |
| HORVU7Hr1G058120 | 5,773195644 | Chlorophyll a-b binding protein, chloroplastic | photosynthesis, light harvesting;photosystem II;chloroplast envelope;photosystem I;regulation of transcription, DNA-templated;response to light stimulus;chloroplast thylakoid membrane;nucleic acid binding;carbohydrate metabolic process;DNA-binding transcription factor activity;metal ion binding;catalytic activity;integral component of membrane;protein-chromophore linkage;chlorophyll binding;carbohydrate binding;plastoglobule; |
| HORVU4Hr1G087310 | 5,708207282 | Acid phosphatase 1 | RNA phosphodiester bond hydrolysis, endonucleolytic;nucleic acid binding;RNA-DNA hybrid ribonuclease activity;dephosphorylation;acid phosphatase activity; |
| HORVU5Hr1G089230 | 5,690483756 | Fatty acyl-CoA reductase | alcohol-forming fatty acyl-CoA reductase activity;lipid metabolic process;integral component of membrane;oxidation-reduction process;fatty-acyl-CoA reductase (alcohol-forming) activity;suberin biosynthetic process;long-chain fatty-acyl-CoA metabolic process; |
| HORVU1Hr1G075160 | 5,685643865 | Phenylcoumaran benzylic ether reductase | phenylpropanoid biosynthetic process;response to cadmium ion;plasma membrane;2'-hydroxyisoflavone reductase activity;cellular response to nitrogen starvation;lignan metabolic process;oxidoreductase activity, acting on NAD(P)H, oxygen as acceptor;response to oxidative stress;cytoplasm;cellular response to sulfur starvation;integral component of membrane;NADPH binding;oxidation-reduction process;cellular response to phosphate starvation; |
| HORVU4Hr1G019430 | 5,556335854 | 0 | regulation of Rho protein signal transduction;DNA repair;DNA packaging complex;membrane docking;cation transmembrane transporter activity;chromosome segregation;Golgi membrane;chromatin organization;DNA packaging;positive regulation of transcription elongation from RNA polymerase II promoter;cell division;ATP binding;transcription factor complex;DNA-binding transcription factor activity;organelle localization;RNA binding;vesicle-mediated transport;flower development;cation transmembrane transport;Wnt signaling pathway;secretion by cell;molecular adaptor activity;protein-DNA complex assembly;Rho guanyl-nucleotide exchange factor activity;visual perception;chromatin;Cul4-RING E3 ubiquitin ligase complex;plasma membrane;cell cycle;nucleus;DNA binding;ubiquitin-protein transferase activity;intracellular organelle lumen;metal ion binding;nucleolar large rRNA transcription by RNA polymerase I;ribosomal large subunit biogenesis;cell differentiation;protein-DNA complex;integral component of membrane;DNA helicase complex;rRNA-containing ribonucleoprotein complex export from nucleus;actin cytoskeleton organization;protein ubiquitination; |
| HORVU1Hr1G085050 | 5,497171033 | Membrane protein-like | metal ion binding;integral component of membrane;oxidation-reduction process;monooxygenase activity; |
| HORVU3Hr1G071770 | 5,446886235 | Putative cellulose synthase (Fragment) | seed trichome differentiation;plant-type secondary cell wall biogenesis;plant-type primary cell wall biogenesis;plant-type cell wall biogenesis;response to water deprivation;trans-Golgi network;mitotic cytokinesis;seed coat development;cellulose biosynthetic process;cell wall organization;defense response to fungus;mucilage biosynthetic process;response to osmotic stress;UDP-glucose metabolic process;ribosome;defense response to bacterium;plasma membrane;DNA binding;nucleus;protein binding;metal ion binding;cell wall;integral component of membrane;cellulose synthase (UDP-forming) activity;cell periphery; |
| HORVU6Hr1G073100 | 5,440734217 | Threonine endopeptidase | aminoacyl-tRNA ligase activity;integral component of membrane;chloroplast part;plastid stroma;plastid envelope;thylakoid;organelle subcompartment; |
| HORVU2Hr1G010670 | 5,440150725 | Ribulose bisphosphate carboxylase small chain | carbohydrate biosynthetic process;photorespiration;photosynthesis;oxidation-reduction process;carbon fixation;monooxygenase activity;ribulose-bisphosphate carboxylase activity;plastid; |
| HORVU5Hr1G016600 | 5,432328868 | Nucleoredoxin | RNA phosphodiester bond hydrolysis, endonucleolytic;zinc ion binding;catalytic activity, acting on a protein;cellular protein modification process;nucleic acid binding;RNA-DNA hybrid ribonuclease activity;ATP binding;integral component of membrane;phosphorylation;carbohydrate binding;phosphotransferase activity, alcohol group as acceptor;kinase activity;intracellular signal transduction; |
| HORVU1Hr1G088930 | 5,39590481 | Chlorophyll a-b binding protein, chloroplastic | photosynthesis, light harvesting;photosystem II;chloroplast envelope;photosystem I;regulation of transcription, DNA-templated;response to light stimulus;chloroplast thylakoid membrane;nucleic acid binding;carbohydrate metabolic process;DNA-binding transcription factor activity;metal ion binding;catalytic activity;integral component of membrane;protein-chromophore linkage;chlorophyll binding;carbohydrate binding;plastoglobule; |
| HORVU0Hr1G032480 | 5,390489047 | Transmembrane protein | cell wall;cell wall organization;extracellular region; |
| HORVU7Hr1G032340 | 5,343925103 | Pectinesterase | extracellular region;nucleolus;maturation of 5.8S rRNA from tricistronic rRNA transcript (SSU-rRNA, 5.8S rRNA, LSU-rRNA);cell wall modification;rRNA (adenine-N6,N6-)-dimethyltransferase activity;nucleoplasm;maturation of LSU-rRNA from tricistronic rRNA transcript (SSU-rRNA, 5.8S rRNA, LSU-rRNA);pectin catabolic process;response to bacterium;plasmodesma;RNA binding;negative regulation of catalytic activity;enzyme inhibitor activity;aspartyl esterase activity;cell wall;ribonucleoprotein complex binding;integral component of membrane;rRNA methylation;pectinesterase activity;preribosome, large subunit precursor; |
| HORVU3Hr1G057860 | 5,338257619 | 0 | ATP binding;cysteine-type endopeptidase inhibitor activity;integral component of membrane;negative regulation of endopeptidase activity;plant-type cell wall organization;protein kinase activity;structural constituent of cell wall;protein phosphorylation; |
| HORVU5Hr1G084230 | 5,245302197 | Plasma membrane intrinsic protein 2 | channel activity;transmembrane transport;water transmembrane transporter activity;vacuole;protein heterotetramerization;plasma membrane;response to water deprivation;water transport;plasmodesma;protein binding;protein homotetramerization;integral component of membrane;protein-containing complex;hydrolase activity; |
| HORVU3Hr1G086160 | 5,233148105 | Laccase | RNA phosphodiester bond hydrolysis, endonucleolytic;nucleic acid binding;lignin catabolic process;RNA-DNA hybrid ribonuclease activity;transmembrane transport;copper ion binding;apoplast;integral component of membrane;oxidation-reduction process;metal ion transmembrane transporter activity;hydroquinone:oxygen oxidoreductase activity;metal ion transport; |
| HORVU4Hr1G087250 | 5,211757742 | 3'-N-debenzoyl-2'-deoxytaxol N-benzoyltransferase | transferase activity, transferring acyl groups other than amino-acyl groups;oxidoreductase activity;oxidation-reduction process; |
| HORVU2Hr1G119050 | 5,206550142 | NADPH-protochlorophyllide oxidoreductase | chlorophyll biosynthetic process;transferase activity, transferring acyl groups;protein domain specific binding;DNA integration;plastid envelope;response to ethylene;nucleic acid binding;chloroplast;metal ion binding;plastid thylakoid membrane;integral component of membrane;photosynthesis;oxidation-reduction process;protochlorophyllide reductase activity; |
| HORVU2Hr1G010690 | 5,144765026 | Ribulose bisphosphate carboxylase small chain | carbohydrate biosynthetic process;photorespiration;photosynthesis;oxidation-reduction process;carbon fixation;monooxygenase activity;ribulose-bisphosphate carboxylase activity;plastid; |
| HORVU5Hr1G047630 | 5,134746184 | TRICHOME BIREFRINGENCE-LIKE 34 | xylan biosynthetic process;chitinase activity;cell wall macromolecule catabolic process;chitin catabolic process;bounding membrane of organelle;integral component of membrane;response to freezing;Golgi apparatus;intracellular organelle part;O-acetyltransferase activity; |
| HORVU7Hr1G072450 | 5,129221294 | MYB transcription factor | positive regulation of secondary metabolite biosynthetic process;regulation of syringal lignin biosynthetic process;nucleus;DNA binding;regulatory region nucleic acid binding;ATP binding;MAPK cascade;DNA-binding transcription factor activity;positive regulation of transcription, DNA-templated;integral component of membrane;cell differentiation;MAP kinase activity;regulation of secondary cell wall biogenesis; |
| HORVU5Hr1G109250 | 5,079599801 | Chlorophyll a-b binding protein, chloroplastic | photosynthesis, light harvesting;photosystem II;chloroplast envelope;photosystem I;regulation of transcription, DNA-templated;response to light stimulus;chloroplast thylakoid membrane;nucleic acid binding;DNA-binding transcription factor activity;metal ion binding;integral component of membrane;protein-chromophore linkage;chlorophyll binding;plastoglobule; |
| HORVU7Hr1G036390 | 5,067870152 | Blue copper | response to aluminum ion;response to zinc ion;seed trichome initiation;response to salt stress;seed trichome elongation;electron transfer activity;response to copper ion;electron transport chain;response to cytokinin;anchored component of plasma membrane;response to auxin;response to abscisic acid;metal ion binding;integral component of membrane;response to mannitol; |
| HORVU7Hr1G083970 | 5,063601964 | NADP-dependent alkenal double bond reductase | oxidoreductase activity;integral component of membrane;oxidation-reduction process;ADP binding; |
| HORVU6Hr1G066460 | 5,044186679 | E3 ubiquitin-protein ligase HERC2 | zinc ion binding; |
| HORVU4Hr1G074520 | 5,037974744 | Amine oxidase | amine metabolic process;copper ion binding;quinone binding;primary amine oxidase activity;integral component of membrane;oxidation-reduction process; |
|  |  |  |  |
| HORVU1Hr1G067080 | -5,001041715 | High molecular mass early light-inducible protein HV58, chloroplastic | positive regulation of seed germination;regulation of chlorophyll biosynthetic process;photosystem II;response to karrikin;photosystem I;cellular response to far red light;cellular response to blue light;response to cold;chloroplast;cellular response to red light;cellular response to UV-A;cellular response to heat;cellular response to high light intensity;integral component of membrane;plastid thylakoid membrane;photosynthesis;photoprotection;response to UV-B; |
| HORVU2Hr1G112120 | -5,023670477 | 0 | transferase activity, transferring acyl groups other than amino-acyl groups; |
| HORVU3Hr1G065770 | -5,130658967 | Bidirectional sugar transporter SWEET | identical protein binding;transmembrane transport;metal ion binding;carbohydrate transport;integral component of membrane;sugar transmembrane transporter activity;plasma membrane; |
| HORVU5Hr1G000620 | -5,166147428 | High molecular mass early light-inducible protein HV58, chloroplastic | positive regulation of seed germination;regulation of chlorophyll biosynthetic process;response to karrikin;photosystem II;photosystem I;cellular response to far red light;cellular response to blue light;response to cold;chloroplast;cellular response to red light;cellular response to UV-A;cellular response to heat;cellular response to high light intensity;integral component of membrane;plastid thylakoid membrane;photosynthesis;photoprotection; |
| HORVU5Hr1G063590 | -5,271728742 | Low molecular mass early light-inducible protein HV60, chloroplastic | organelle envelope;chloroplast;integral component of membrane;thylakoid membrane;plastid part;organelle membrane;organelle subcompartment; |
| HORVU2Hr1G124970 | -5,291222898 | Peroxidase | mucilage extrusion from seed coat;extracellular region;plant-type cell wall;ribosome;vacuole;cell wall polysaccharide catabolic process;hydrogen peroxide catabolic process;heme binding;translation;peroxidase activity;response to oxidative stress;plant-type cell wall modification;plasmodesma;structural constituent of ribosome;metal ion binding;lignin biosynthetic process;membrane;cellular oxidant detoxification;integral component of membrane;oxidation-reduction process; |
| HORVU7Hr1G087270 | -5,337467617 | MACPF domain-containing protein isoform A | plant-type hypersensitive response;plasma membrane; |
| HORVU2Hr1G120660 | -5,492082279 | L-type lectin-domain containing receptor kinase IV.2 | ATP binding;integral component of membrane;protein kinase activity;carbohydrate binding;protein phosphorylation; |
| HORVU0Hr1G023360 | -5,547451372 | High molecular mass early light-inducible protein HV58, chloroplastic | positive regulation of seed germination;regulation of chlorophyll biosynthetic process;response to karrikin;photosystem II;photosystem I;cellular response to far red light;cellular response to blue light;response to cold;chloroplast;cellular response to red light;cellular response to UV-A;cellular response to heat;cellular response to high light intensity;integral component of membrane;plastid thylakoid membrane;photosynthesis;photoprotection; |
| HORVU0Hr1G027960 | -5,556869747 | p21-activated protein kinase-interacting protein 1-like | cell population proliferation;catalytic activity, acting on a protein;roof of mouth development;regulation of signal transduction by p53 class mediator;endoplasmic reticulum;nucleolus;Cul4-RING E3 ubiquitin ligase complex;negative regulation of signal transduction;ribosomal large subunit biogenesis;integral component of membrane;oxidoreductase activity, acting on a sulfur group of donors, disulfide as acceptor;oxidation-reduction process;phosphorylation;kinase activity;intramolecular oxidoreductase activity, transposing S-S bonds; |
| HORVU2Hr1G108400 | -5,565745559 | Oligopeptide transporter | transmembrane transport;integral component of membrane;vacuolar membrane; |
| HORVU4Hr1G063190 | -5,657423598 | Early light-inducible protein ELIP | positive regulation of seed germination;regulation of chlorophyll biosynthetic process;photosystem II;response to karrikin;photosystem I;cellular response to far red light;cellular response to blue light;response to cold;chloroplast;cellular response to red light;cellular response to UV-A;cellular response to heat;cellular response to high light intensity;integral component of membrane;plastid thylakoid membrane;photosynthesis;photoprotection;response to UV-B; |
| HORVU2Hr1G000080 | -5,666155798 | probable protein phosphatase 2C 75 | cation binding;protein phosphorylation;negative regulation of protein kinase activity;response to water deprivation;hormone-mediated signaling pathway;ATP binding;response to cold;response to heat;regulation of stomatal movement;cellular response to lipid;cellular response to alcohol;cytosol;protein kinase binding;positive regulation of seed germination;protein dephosphorylation;glycolytic process;response to high light intensity;response to osmotic stress;negative regulation of photosynthesis, light reaction;protein kinase activity;plasma membrane;negative regulation of abscisic acid-activated signaling pathway;protein serine/threonine phosphatase activity;nucleus;pyruvate kinase activity;response to abscisic acid;metal ion binding;integral component of membrane;negative regulation of transforming growth factor beta receptor signaling pathway;cellular response to acid chemical; |
| HORVU1Hr1G072100 | -5,682071098 | 0 | nucleic acid binding;ATP binding;microtubule motor activity;integral component of membrane;response to chitin;microtubule-based movement;microtubule binding;phosphatidylinositol dephosphorylation; |
| HORVU4Hr1G024270 | -5,715382905 | RNA recognition motif domain | RNA binding;protein binding;mRNA transport;nucleoplasm; |
| HORVU1Hr1G087740 | -5,725316218 | Early light-inducible protein ELIP | organelle envelope;chloroplast;integral component of membrane;thylakoid membrane;plastid part;organelle membrane;organelle subcompartment; |
| HORVU4Hr1G066810 | -5,765072761 | High molecular mass early light-inducible protein HV58, chloroplastic | positive regulation of seed germination;regulation of chlorophyll biosynthetic process;photosystem II;response to karrikin;photosystem I;thylakoid membrane;cellular response to far red light;plastid part;organelle membrane;organelle envelope;cellular response to blue light;response to cold;chloroplast;cellular response to red light;cellular response to UV-A;cellular response to heat;cellular response to high light intensity;integral component of membrane;photosynthesis;photoprotection;response to UV-B;organelle subcompartment; |
| HORVU2Hr1G091880 | -5,779157164 | Vacuolar-processing enzyme beta-isozyme | nucleus;protein catabolic process;cellular macromolecule catabolic process;proteolysis;protein storage vacuole organization;integral component of membrane;cellular protein metabolic process;protein storage vacuole;protein maturation;peptidase activity;nutrient reservoir activity; |
| HORVU4Hr1G019130 | -5,832938121 | AP complex subunit sigma | DNA repair;protein disulfide oxidoreductase activity;FMN binding;electron transfer activity;protein transport;DNA recombination;serine-type endopeptidase activity;telomere maintenance;cysteine-type peptidase activity;ubiquitin-like modifier activating enzyme activity;cell redox homeostasis;electron transport chain;intracellular protein transport;nucleic acid binding;membrane coat;ATP binding;DNA helicase activity;vesicle-mediated transport;DNA duplex unwinding;proteolysis;membrane;integral component of membrane;organelle localization by membrane tethering;secretion by cell; |
| HORVU1Hr1G004550 | -5,927458443 | Envelope glycoprotein | transferase activity, transferring glycosyl groups;protein glycosylation;fucose biosynthetic process;salicylic acid mediated signaling pathway;photosynthetic electron transport in photosystem I;chloroplast thylakoid membrane;xyloglucan biosynthetic process;unidimensional cell growth;intrinsic component of Golgi membrane;trans-Golgi network;endosome;integral component of membrane;oxidoreductase activity, acting on iron-sulfur proteins as donors;endomembrane system organization; |
| HORVU3Hr1G002520 | -6,042134392 | Rust resistance kinase Lr10 | ATP binding;catalytic activity, acting on a protein;integral component of membrane;protein phosphorylation;phosphotransferase activity, alcohol group as acceptor;kinase activity;polysaccharide binding; |
| HORVU4Hr1G066800 | -6,093511831 | High molecular mass early light-inducible protein HV58, chloroplastic | positive regulation of seed germination;regulation of chlorophyll biosynthetic process;photosystem II;response to karrikin;photosystem I;thylakoid membrane;cellular response to far red light;plastid part;organelle membrane;organelle envelope;cellular response to blue light;response to cold;chloroplast;cellular response to red light;cellular response to UV-A;cellular response to heat;cellular response to high light intensity;integral component of membrane;photosynthesis;photoprotection;response to UV-B;organelle subcompartment; |
| HORVU5Hr1G063620 | -6,196916768 | Low molecular mass early light-inducible protein HV90, chloroplastic | organelle envelope;chloroplast;integral component of membrane;thylakoid membrane;plastid part;organelle membrane;organelle subcompartment; |
| HORVU6Hr1G083960 | -6,309777987 | Dehydrin | response to abscisic acid;cytosol;cold acclimation;response to water; |
| HORVU4Hr1G039680 | -6,315593358 | 0 | protein disulfide oxidoreductase activity;response to salt stress;electron transfer activity;electron transport chain;phosphopyruvate hydratase complex;response to light stimulus;phosphopyruvate hydratase activity;magnesium ion binding;response to cold;cell;chloroplast;plasmodesma;copper ion binding;apoplast;malate dehydrogenase activity;oxidoreductase activity, acting on the CH-OH group of donors, NAD or NADP as acceptor;glycolytic process;response to cadmium ion;cell redox homeostasis;mitochondrial envelope;plasma membrane;DNA binding;nucleus;organelle outer membrane;response to abscisic acid;malate metabolic process;oxidation-reduction process; |
| HORVU5Hr1G080440 | -6,505456599 | C-repeat binding factor roughrider | nucleus;DNA binding;DNA-binding transcription factor activity;regulation of transcription, DNA-templated; |
| HORVU5Hr1G063570 | -7,413597404 | Low molecular mass early light-inducible protein HV90, chloroplastic | organelle envelope;chloroplast;integral component of membrane;thylakoid membrane;plastid part;organelle membrane;organelle subcompartment; |
| HORVU0Hr1G030110 | -7,515103696 | 0 | nucleus;zinc ion binding;regulation of flower development;circadian rhythm;protein binding, bridging; |
| HORVU5Hr1G063660 | -8,527465232 | Low molecular mass early light-inducible protein HV90, chloroplastic | organelle envelope;chloroplast;integral component of membrane;thylakoid membrane;plastid part;organelle membrane;organelle subcompartment; |
| HORVU7Hr1G019870 | -8,860661427 | Pre-mRNA-splicing factor | ATPase activity;RNA splicing;response to salt stress;mRNA processing;inorganic phosphate transmembrane transporter activity;ATP binding;precatalytic spliceosome;membrane;response to lithium ion;integral component of membrane;phosphorylation;kinase activity;phosphate ion transmembrane transport; |
|  |  |  |  |
| **B.** Genes highly expressed at BW084 (log2FC  > 5 (-5) ) but undetectable in BW (comparison of de-acclimated plants to cold acclimated plants) | | | |
| HORVU | **log2FC** | **annotation** | **GO terms** |
| HORVU2Hr1G059150 | 8,025042879 | Outer envelope membrane protein 7 | integral component of membrane;vacuolar membrane; |
| HORVU5Hr1G095270 | 6,963258368 | Atrophin-1 like | integral component of membrane;hydrolase activity; |
| HORVU6Hr1G066460 | 6,871872069 | E3 ubiquitin-protein ligase HERC2 | zinc ion binding; |
| HORVU3Hr1G023780 | 6,425121308 | Shikimate O-hydroxycinnamoyltransferase | transferase activity, transferring acyl groups other than amino-acyl groups; |
| HORVU5Hr1G066250 | 6,068014082 | Vacuolar-processing enzyme gamma-isozyme | nucleic acid binding;proteolysis;DNA integration;protein maturation;peptidase activity;cellular protein catabolic process;vacuole; |
| HORVU5Hr1G065380 | 5,769102376 | WRKY transcription factor | seed trichome initiation;regulation of transcription, DNA-templated;sequence-specific DNA binding;response to fungus;response to salicylic acid;photosynthetic electron transport in photosystem I;negative regulation of defense response to bacterium;cotyledon development;chloroplast thylakoid membrane;nucleus;DNA-binding transcription factor activity;response to bacterium;anther development;sepal development;response to chitin;stigma development;NAD(P)H dehydrogenase complex (plastoquinone); |
| HORVU6Hr1G009420 | 5,711074967 | Peroxidase | peroxidase activity;response to oxidative stress;metal ion binding;integral component of membrane;cellular oxidant detoxification;oxidation-reduction process;extracellular region;hydrogen peroxide catabolic process;heme binding; |
| HORVU5Hr1G095580 | 5,670538241 | Senescence-specific cysteine protease | response to fructose;leaf senescence;floral organ senescence;endopeptidase activity;cysteine-type peptidase activity;plant-type vacuole;cellular protein catabolic process;plant-type hypersensitive response;lytic vacuole;response to cytokinin;response to ethylene;response to sucrose;response to auxin;defense response to fungus, incompatible interaction;response to gibberellin;extracellular space;response to glucose;response to abscisic acid;stress response to copper ion;proteolysis;integral component of membrane;programmed cell death involved in cell development;response to UV-B; |
| HORVU7Hr1G083970 | 5,555393445 | NADP-dependent alkenal double bond reductase | oxidoreductase activity;integral component of membrane;oxidation-reduction process;ADP binding; |
| HORVU2Hr1G024280 | 5,504425445 | Peroxidase | peroxidase activity;response to oxidative stress;metal ion binding;integral component of membrane;cellular oxidant detoxification;oxidation-reduction process;extracellular region;hydrogen peroxide catabolic process;heme binding; |
| HORVU7Hr1G078700 | 5,495790047 | Molybdate transporter like | organelle envelope;molybdate ion transmembrane transporter activity;mitochondrion;integral component of membrane;endomembrane system;vacuole;organelle membrane;molybdate ion transport;plasma membrane; |
| HORVU6Hr1G008780 | 5,431390966 | Cysteine endopeptidase | Sertoli cell differentiation;external side of plasma membrane;peptide binding;decidualization;fusome;cellular protein catabolic process;perikaryon;neuron projection;protein-containing complex binding;nerve development;vacuolar lumen;response to glucose;apical part of cell;microvillus;autophagic cell death;apoplast;response to glucocorticoid;multicellular organism aging;antigen processing and presentation of exogenous peptide antigen via MHC class II;kininogen binding;regulation of keratinocyte differentiation;2-alkenal reductase [NAD(P)] activity;secretory granule;spermatogenesis;extracellular matrix disassembly;endopeptidase activity;digestion;cysteine-type peptidase activity;aminopeptidase activity;cellular response to starvation;lysosome;extracellular space;polysaccharide catabolic process;proteolysis;response to odorant;integral component of membrane;response to gonadotropin;oxidation-reduction process; |
| HORVU1Hr1G068170 | 5,193193133 | Tubulin beta chain | cytoskeleton organization;response to salt stress;seed trichome differentiation;developmental growth involved in morphogenesis;arginyl-tRNA aminoacylation;mitotic cell cycle;microtubule;microtubule-based process;protein retention in ER lumen;GTP binding;response to cold;ATP binding;response to abiotic stimulus;arginine-tRNA ligase activity;tubulin complex;GTPase activity;intracellular membrane-bounded organelle;transferase activity, transferring glycosyl groups;cell morphogenesis involved in differentiation;cell morphogenesis;structural constituent of cytoskeleton;cell development;ER retention sequence binding;plasma membrane;response to stress;protein binding;cytoplasm;BRISC complex;bounding membrane of organelle;cell wall;integral component of membrane;endomembrane system;BRCA1-A complex;cell growth;whole membrane; |
| HORVU5Hr1G095540 | 5,151684111 | Calcium-activated outward-rectifying potassium channel, putative | cellular potassium ion homeostasis;integral component of plasma membrane;plant-type vacuole membrane;potassium ion transmembrane transport;calcium ion binding;inward rectifier potassium channel activity;stabilization of membrane potential;potassium ion-transporting ATPase complex;potassium ion leak channel activity; |
| HORVU7Hr1G089480 | 5,107108809 | Peroxidase | extracellular region;plant-type cell wall;hydrogen peroxide catabolic process;heme binding;peroxidase activity;response to oxidative stress;defense response to bacterium, incompatible interaction;plasmodesma;metal ion binding;cytoplasm;cellular oxidant detoxification;integral component of membrane;oxidation-reduction process; |
|  |  |  |  |
| HORVU5Hr1G077390 | -5,159462121 | BHLH transcription factor | nucleus;ATP binding;DNA-binding transcription factor activity;transcription, DNA-templated;integral component of membrane;regulation of transcription, DNA-templated;protein dimerization activity;sequence-specific DNA binding;protein kinase activity;protein phosphorylation;positive regulation of shade avoidance;polysaccharide binding; |
| HORVU6Hr1G095260 | -5,223927936 | Rust resistance kinase Lr10 | ATP binding;protein tyrosine kinase activity;integral component of membrane;peptidyl-tyrosine modification;protein serine/threonine kinase activity;protein phosphorylation;carbohydrate binding; |
| HORVU3Hr1G012080 | -5,396062732 | Protein kinase/ transmembrane receptor protein serine/threonine kinase | ATP binding;integral component of membrane;protein kinase activity;protein phosphorylation; |
| HORVU2Hr1G099820 | -5,407810224 | Cold-responsive protein Wcs19-B | lipid transport;response to heat;iron ion binding;electron transfer activity;integral component of membrane;response to desiccation;lipid binding;lipoprotein metabolic process;electron transport chain;extracellular region;hyperosmotic response;heme binding; |
| HORVU5Hr1G113080 | -5,415097457 | C2H2 transcription factor | nucleic acid binding;ATP binding;microtubule motor activity;clathrin coat assembly;mitochondrion;clathrin binding;clathrin-coated vesicle;microtubule-based movement;1-phosphatidylinositol binding;microtubule binding; |
| HORVU5Hr1G113120 | -5,415097457 | C2H2 transcription factor | nucleic acid binding;ATP binding;microtubule motor activity;clathrin coat assembly;mitochondrion;clathrin binding;clathrin-coated vesicle;microtubule-based movement;1-phosphatidylinositol binding;microtubule binding; |
| HORVU7Hr1G046430 | -5,511855239 | Rhoptry neck protein 2 (Fragment) | DNA repair;determination of adult lifespan;protein transport;snoRNA processing;helicase activity;Golgi membrane;RNA splicing, via transesterification reactions with bulged adenosine as nucleophile;polytene chromosome;5'-3' exoribonuclease activity;exoribonuclease II activity;RNA binding;positive regulation of transcription, DNA-templated;nematode larval development;protein localization to peroxisome;cytosol;nuclear-transcribed mRNA catabolic process, exonucleolytic, 5'-3';negative regulation of protein metabolic process;RNA phosphodiester bond hydrolysis, exonucleolytic;zinc ion binding;positive regulation of DNA topoisomerase (ATP-hydrolyzing) activity;retrograde vesicle-mediated transport, Golgi to endoplasmic reticulum;protein-DNA complex assembly;thiamine metabolic process;chromatin;hyperosmotic response;nucleus;regulation of mRNA processing;peroxisomal membrane transport;protein ubiquitination;DNA replication;extracellular region;ATP binding;termination of RNA polymerase II transcription, poly(A)-coupled;response to heat;pseudouridine synthesis;dosage compensation;nuclear polyadenylation-dependent rRNA catabolic process;termination of RNA polymerase II transcription, exosome-dependent;DNA duplex unwinding;positive regulation of heterochromatin assembly;regulation of cellular amide metabolic process;negative regulation of DNA-templated transcription, elongation;regulation of cellular protein metabolic process;phosphorylation;COPI vesicle coat;cytoplasmic ribonucleoprotein granule;thiamine-containing compound biosynthetic process;DNA-dependent ATPase activity;catalytic activity, acting on DNA;transmembrane transport;X chromosome;germ plasm;DNA recombination;phosphomethylpyrimidine kinase activity;cleavage in ITS2 between 5.8S rRNA and LSU-rRNA of tricistronic rRNA transcript (SSU-rRNA, 5.8S rRNA, LSU-rRNA);male courtship behavior, veined wing generated song production;chromatin binding;pseudouridine synthase activity;mRNA processing;regulation of transcription by RNA polymerase II;negative regulation of phosphorylation;posttranscriptional regulation of gene expression;establishment of protein localization to organelle;peroxisome;regulatory region nucleic acid binding;DNA binding;RNA-DNA hybrid ribonuclease activity;ubiquitin-protein transferase activity;axon extension;intracellular organelle lumen;lipid metabolic process;integral component of membrane;nuclear mRNA surveillance;protein acetyltransferase complex; |
| HORVU5Hr1G071940 | -5,54505547 | Cyanohydrin beta-glucosyltransferase | UDP-glycosyltransferase activity;dhurrin biosynthetic process;transferase activity, transferring hexosyl groups;tRNA (guanine-N7-)-methyltransferase activity;glycolytic process;cytoplasmic part;membrane part;organelle membrane;intracellular organelle part;intracellular membrane-bounded organelle;phosphoglycerate kinase activity;nuclear outer membrane-endoplasmic reticulum membrane network;tRNA (guanine-N7)-methylation; |
| HORVU4Hr1G054420 | -5,560605241 | MYB transcription factor | nucleus;DNA binding;regulatory region nucleic acid binding;plant epidermis development;DNA-binding transcription factor activity;cell differentiation;integral component of membrane;regulation of transcription, DNA-templated;seed development;plant-type secondary cell wall biogenesis;cellular process involved in reproduction in multicellular organism; |
| HORVU1Hr1G024180 | -5,566010755 | Arabinogalactan protein 2 | somatic embryogenesis;response to salt stress;cell morphogenesis involved in differentiation;seed trichome differentiation;double-stranded DNA binding;regulation of transcription, DNA-templated;cell adhesion;plant-type secondary cell wall biogenesis;response to cytokinin;plasma membrane;response to auxin;response to gibberellin;unidimensional cell growth;response to abscisic acid;regulation of protein localization to cell surface;intrinsic component of membrane; |
| HORVU5Hr1G063570 | -5,732863861 | Low molecular mass early light-inducible protein HV90, chloroplastic | organelle envelope;chloroplast;integral component of membrane;thylakoid membrane;plastid part;organelle membrane;organelle subcompartment; |
| HORVU2Hr1G099370 | -5,736808485 | Bifunctional inhibitor/lipid-transfer protein/seed storage 2S albumin superfamily protein isoform 1 | GTPase activator activity;DNA integration;actin filament organization;structural constituent of cell wall;extracellular region;protein phosphorylation;positive regulation of GTPase activity;chloroplast thylakoid membrane;GTP binding;ATP binding;plasmodesma;GTPase activity;Rac GTPase binding;cytosol;potassium ion transmembrane transport;signal transduction;DNA recombination;protein serine/threonine kinase activity;nucleic acid binding;nucleus;calmodulin binding;regulation of actin cytoskeleton organization;cell wall;integral component of membrane;small conductance calcium-activated potassium channel activity;calcium-ion regulated exocytosis; |
| HORVU2Hr1G000080 | -5,769087391 | probable protein phosphatase 2C 75 | cation binding;protein phosphorylation;negative regulation of protein kinase activity;response to water deprivation;hormone-mediated signaling pathway;ATP binding;response to cold;response to heat;regulation of stomatal movement;cellular response to lipid;cellular response to alcohol;cytosol;protein kinase binding;positive regulation of seed germination;protein dephosphorylation;glycolytic process;response to high light intensity;response to osmotic stress;negative regulation of photosynthesis, light reaction;protein kinase activity;plasma membrane;negative regulation of abscisic acid-activated signaling pathway;protein serine/threonine phosphatase activity;nucleus;pyruvate kinase activity;response to abscisic acid;metal ion binding;integral component of membrane;negative regulation of transforming growth factor beta receptor signaling pathway;cellular response to acid chemical; |
| HORVU6Hr1G083960 | -5,803131418 | Dehydrin | response to abscisic acid;cytosol;cold acclimation;response to water; |
| HORVU2Hr1G091880 | -5,835230133 | Vacuolar-processing enzyme beta-isozyme | nucleus;protein catabolic process;cellular macromolecule catabolic process;proteolysis;protein storage vacuole organization;integral component of membrane;cellular protein metabolic process;protein storage vacuole;protein maturation;peptidase activity;nutrient reservoir activity; |
| HORVU1Hr1G067080 | -6,082711273 | High molecular mass early light-inducible protein HV58, chloroplastic | positive regulation of seed germination;regulation of chlorophyll biosynthetic process;photosystem II;response to karrikin;photosystem I;cellular response to far red light;cellular response to blue light;response to cold;chloroplast;cellular response to red light;cellular response to UV-A;cellular response to heat;cellular response to high light intensity;integral component of membrane;plastid thylakoid membrane;photosynthesis;photoprotection;response to UV-B; |
| HORVU3Hr1G073910 | -6,10294936 | Glycerophosphoryl diester phosphodiesterase | glycerol metabolic process;lipid metabolic process;protein transport;integral component of membrane;cytosol;phosphoric diester hydrolase activity;vacuole;exocytosis;plasma membrane;exocyst; |
| HORVU0Hr1G023360 | -6,117689486 | High molecular mass early light-inducible protein HV58, chloroplastic | positive regulation of seed germination;regulation of chlorophyll biosynthetic process;response to karrikin;photosystem II;photosystem I;cellular response to far red light;cellular response to blue light;response to cold;chloroplast;cellular response to red light;cellular response to UV-A;cellular response to heat;cellular response to high light intensity;integral component of membrane;plastid thylakoid membrane;photosynthesis;photoprotection; |
| HORVU5Hr1G080440 | -7,208013385 | C-repeat binding factor roughrider | nucleus;DNA binding;DNA-binding transcription factor activity;regulation of transcription, DNA-templated; |
| HORVU0Hr1G030110 | -7,41263787 | 0 | nucleus;zinc ion binding;regulation of flower development;circadian rhythm;protein binding, bridging; |
| HORVU5Hr1G063660 | -8,474952902 | Low molecular mass early light-inducible protein HV90, chloroplastic | organelle envelope;chloroplast;integral component of membrane;thylakoid membrane;plastid part;organelle membrane;organelle subcompartment; |
|  |  |  |  |
| **C.** Genes highly expressed at BW312 (log2FC  > 5 (-5) ) but undetectable in BW (comparison of de-acclimated plants to cold acclimated plants) | | | |
| **HORVU** | **log2FC** | **annotation** | **GO terms** |
| HORVU2Hr1G059150 | 9,916948425 | Outer envelope membrane protein 7 | integral component of membrane;vacuolar membrane; |
| HORVU7Hr1G002360 | 8,951382692 | Aspartic proteinase nepenthesin-1 | aspartic-type endopeptidase activity;protein catabolic process;proteolysis;integral component of membrane; |
| HORVU5Hr1G095270 | 8,466098138 | Atrophin-1 like | integral component of membrane;hydrolase activity; |
| HORVU5Hr1G066250 | 8,415275671 | Vacuolar-processing enzyme gamma-isozyme | nucleic acid binding;proteolysis;DNA integration;protein maturation;peptidase activity;cellular protein catabolic process;vacuole; |
| HORVU6Hr1G082360 | 8,395124002 | 0 | DNA packaging complex;response to salt stress;serine-type carboxypeptidase activity;protein self-association;non-membrane-bounded organelle;membrane-bounded organelle;response to water deprivation;response to cold;response to heat;DNA-binding transcription factor activity;positive regulation of transcription, DNA-templated;pollen maturation;protein homodimerization activity;response to high light intensity;unfolded protein binding;response to hydrogen peroxide;organelle part;protein folding;intracellular part;seed germination;nucleosome assembly;DNA binding;abscisic acid-activated signaling pathway;proteolysis;protein-DNA complex;protein complex oligomerization; |
| HORVU1Hr1G067980 | 8,345325156 | Dr1-associated corepressor | nucleus;transcription coregulator activity;protein heterodimerization activity;integral component of membrane;regulation of nucleic acid-templated transcription; |
| HORVU3Hr1G032370 | 7,655984343 | Isoflavone reductase-IRL-like protein | coenzyme binding;structural constituent of ribosome;catalytic activity;ribosome;translation; |
| HORVU6Hr1G092540 | 7,522261287 | Subtilisin-like protease | proteolysis;serine-type endopeptidase activity; |
| HORVU5Hr1G106010 | 7,27960928 | Pathogenesis related protein-1 | integral component of membrane;extracellular region; |
| HORVU5Hr1G087940 | 7,226726591 | Mediator of RNA polymerase II transcription subunit 8 | 0 |
| HORVU5Hr1G106480 | 7,173142169 | 0 | nucleus;DNA binding;DNA-binding transcription factor activity;protein homodimerization activity;negative regulation of transcription, DNA-templated;integral component of membrane;ADP binding;cellular response to molecule of bacterial origin;negative regulation of immune response;defense response; |
| HORVU7Hr1G051310 | 6,625856968 | 0 | carbohydrate metabolic process;extracellular space;cellular macromolecule metabolic process;macromolecule catabolic process;integral component of membrane;response to fungus;hydrolase activity, hydrolyzing O-glycosyl compounds;defense response;cellular catabolic process;anchored component of plasma membrane; |
| HORVU2Hr1G107190 | 6,597523271 | Putative LRR receptor-like serine/threonine-protein kinase | ATP binding;integral component of membrane;protein kinase activity;protein phosphorylation; |
| HORVU1Hr1G060880 | 6,595193366 | 0 | RNA phosphodiester bond hydrolysis, endonucleolytic;zinc ion binding;cellular aldehyde metabolic process;chloroplast envelope;methionyl-tRNA aminoacylation;regulation of transcription, DNA-templated;response to water deprivation;aldehyde dehydrogenase (NAD) activity;copper ion transport;RNA-DNA hybrid ribonuclease activity;DNA binding;nucleus;ATP binding;mitochondrial intermembrane space;DNA-binding transcription factor activity;response to abscisic acid;methionine-tRNA ligase activity;integral component of membrane;oxidation-reduction process;copper chaperone activity;aldehyde dehydrogenase (NADP+) activity; |
| HORVU3Hr1G115420 | 6,512317184 | 4-hydroxyphenylacetaldehyde oxime monooxygenase | endoplasmic reticulum membrane;dhurrin biosynthetic process;oxidoreductase activity, acting on paired donors, with incorporation or reduction of molecular oxygen;iron ion binding;integral component of membrane;oxidation-reduction process;monooxygenase activity;heme binding; |
| HORVU5Hr1G112930 | 6,507423496 | Zinc finger protein 1 | nucleic acid binding;mitochondrion; |
| HORVU2Hr1G002700 | 6,464702412 | L-type lectin-domain containing receptor kinase IX.1 | molecular transducer activity;protein transport;protein kinase activity;regulation of apoptotic process;periplasmic space;transmembrane receptor protein serine/threonine kinase signaling pathway;protein phosphorylation;defense response to bacterium;plasma membrane;ATP binding;defense response to oomycetes;integral component of membrane;peptidyl-tyrosine modification;regulation of cell cycle;carbohydrate binding; |
| HORVU1Hr1G001220 | 6,340748179 | Cysteine-rich receptor-like protein kinase 25 | GTP binding;ATP binding;recognition of pollen;GTPase activity;dihydroorotase activity;integral component of membrane;protein kinase activity;protein phosphorylation;plasma membrane;pyrimidine nucleobase biosynthetic process; |
| HORVU2Hr1G074200 | 6,306754857 | 0 | Kibra-Ex-Mer complex;ATPase activity;postsynaptic density;regulation of response to stress;animal organ development;structural constituent of cell wall;negative regulation of biological process;reproductive system development;regulation of cellular process;RNA binding;protein heterodimerization activity;phosphotransferase activity, alcohol group as acceptor;axon;intracellular transport;peripheral nervous system development;microtubule cytoskeleton organization;response to sodium arsenite;DNA-binding transcription factor activity, RNA polymerase II-specific;cytoplasmic side of apical plasma membrane;nucleus;cell-cell junction;positive regulation of signaling;central nervous system development;metal ion binding;visual system development;signaling;neurofibrillary tangle;anatomical structure morphogenesis;columnar/cuboidal epithelial cell differentiation;regulation of nucleosome density;RNA metabolic process;vesicle-mediated transport;response to leukemia inhibitory factor;regulation of intracellular transport;epithelial cell migration;catalytic activity, acting on a protein;phosphatidylinositol binding;plant-type cell wall organization;chromatin binding;macromolecule modification;heme binding;Schaffer collateral - CA1 synapse;DNA binding;cytoskeleton;transcription corepressor activity;mitochondrion;cellular response to stimulus;oxidation-reduction process;regulation of primary metabolic process;organic substance transport;female gamete generation;apicolateral plasma membrane;plasma membrane bounded cell projection organization;head development;sulfotransferase activity;Golgi membrane;response to axon injury;regulation of nitrogen compound metabolic process;developmental process involved in reproduction;regulation of imaginal disc growth;carbohydrate biosynthetic process;activating transcription factor binding;toxic substance binding;neuron differentiation;neurofilament bundle assembly;DNA metabolic process;chromatin;hydrogen peroxide catabolic process;actin binding;supramolecular fiber;ATP-dependent chromatin remodeling;regulation of gene expression;DNA helicase complex;gene expression;nucleobase-containing compound biosynthetic process;protein metabolic process;myelin sheath;hydrolase activity, acting on acid phosphorus-nitrogen bonds;cell communication;perikaryon;positive regulation of response to stimulus;ATP binding;response to oxidative stress;detoxification;regulation of macromolecule biosynthetic process;macromolecule localization;regeneration;phosphorylation;regulation of response to nutrient levels;cellular macromolecule biosynthetic process;neurofilament cytoskeleton organization;structural constituent of postsynaptic intermediate filament cytoskeleton;cell development;postsynaptic intermediate filament cytoskeleton organization;amide transport;cellular process involved in reproduction in multicellular organism;peroxidase activity;intracellular organelle lumen;integral component of membrane;protein binding, bridging;kinase activity;enzyme binding; |
| HORVU5Hr1G072520 | 6,274334103 | Reticuline oxidase | FAD binding;oxidoreductase activity;oxidation-reduction process; |
| HORVU5Hr1G065380 | 6,213789369 | WRKY transcription factor | seed trichome initiation;regulation of transcription, DNA-templated;sequence-specific DNA binding;response to fungus;response to salicylic acid;photosynthetic electron transport in photosystem I;negative regulation of defense response to bacterium;cotyledon development;chloroplast thylakoid membrane;nucleus;DNA-binding transcription factor activity;response to bacterium;anther development;sepal development;response to chitin;stigma development;NAD(P)H dehydrogenase complex (plastoquinone); |
| HORVU7Hr1G113030 | 6,203345759 | Patatin | serine-type endopeptidase inhibitor activity;lipid catabolic process;negative regulation of endopeptidase activity;extracellular region;hydrolase activity; |
| HORVU1Hr1G021680 | 6,200525809 | Indolin-2-one monooxygenase | oxidoreductase activity, acting on paired donors, with incorporation or reduction of molecular oxygen;iron ion binding;integral component of membrane;oxidation-reduction process;monooxygenase activity;heme binding; |
| HORVU0Hr1G013470 | 6,136824486 | Serine carboxypeptidase-like 19 | proteolysis;integral component of membrane;serine-type carboxypeptidase activity; |
| HORVU7Hr1G078700 | 6,08252057 | Molybdate transporter like | organelle envelope;molybdate ion transmembrane transporter activity;mitochondrion;integral component of membrane;endomembrane system;vacuole;organelle membrane;molybdate ion transport;plasma membrane; |
| HORVU0Hr1G002880 | 6,052508929 | Putative zinc finger, RING/FYVE/PHD-type | ubiquitin protein ligase activity;cytoplasm;ligase activity;protein ubiquitination; |
| HORVU3Hr1G032370 | 6,013073745 | Integrase (Fragment) | nucleic acid binding;nucleic acid phosphodiester bond hydrolysis;coenzyme binding;catalytic activity;exonuclease activity;DNA integration;integral component of membrane; |
| HORVU2Hr1G009950 | 5,985609204 | Wall-associatewrkysd kinase | cGMP biosynthetic process;guanylate cyclase activity;protein kinase activity;response to fungus;response to salicylic acid;protein phosphorylation;defense response;plasma membrane;translation;ATP binding;nucleus;response to bacterium;transmembrane signaling receptor activity;nuclear export;structural constituent of ribosome;calcium ion binding;integral component of membrane;peptidyl-tyrosine modification;large ribosomal subunit;polysaccharide binding; |
| HORVU0Hr1G035320 | 5,850719356 | 0 | zinc ion binding;carbonate dehydratase activity;ligase activity; |
| HORVU5Hr1G095580 | 5,848407429 | Senescence-specific cysteine protease | response to fructose;leaf senescence;floral organ senescence;endopeptidase activity;cysteine-type peptidase activity;plant-type vacuole;cellular protein catabolic process;plant-type hypersensitive response;lytic vacuole;response to cytokinin;response to ethylene;response to sucrose;response to auxin;defense response to fungus, incompatible interaction;response to gibberellin;extracellular space;response to glucose;response to abscisic acid;stress response to copper ion;proteolysis;integral component of membrane;programmed cell death involved in cell development;response to UV-B; |
| HORVU6Hr1G014880 | 5,810660072 | Putative LRR receptor-like serine/threonine-protein kinase | ATP binding;oxidoreductase activity;integral component of membrane;protein kinase activity;oxidation-reduction process;protein phosphorylation; |
| HORVU3Hr1G105480 | 5,776947611 | 0 | carbohydrate metabolic process;DNA repair;ATP binding;extracellular space;DNA-dependent ATPase activity;integral component of membrane;hydrolase activity, hydrolyzing O-glycosyl compounds;defense response;anchored component of plasma membrane;single-stranded DNA binding; |
| HORVU3Hr1G105420 | 5,771636474 | Glucan endo-1,3-beta-glucosidase, acidic isoform | carbohydrate metabolic process;extracellular space;cellular macromolecule metabolic process;macromolecule catabolic process;integral component of membrane;response to fungus;hydrolase activity, hydrolyzing O-glycosyl compounds;defense response;cellular catabolic process;anchored component of plasma membrane; |
| HORVU5Hr1G115870 | 5,764082832 | Cell number regulator 9 | response to oxidative stress;pollen sperm cell differentiation;integral component of membrane;plasma membrane; |
| HORVU6Hr1G050750 | 5,636270462 | Cellulose synthase | trans-Golgi network;plasmodesma;endosome;metal ion binding;integral component of membrane;cellulose synthase (UDP-forming) activity;cellulose biosynthetic process;cell wall organization;plant-type secondary cell wall biogenesis;plasma membrane;plant-type primary cell wall biogenesis; |
| HORVU6Hr1G009420 | 5,620958746 | Peroxidase | peroxidase activity;response to oxidative stress;metal ion binding;integral component of membrane;cellular oxidant detoxification;oxidation-reduction process;extracellular region;hydrogen peroxide catabolic process;heme binding; |
| HORVU3Hr1G105560 | 5,614630185 | Beta-glucanase | DNA-dependent ATPase activity;zinc ion binding;drug binding;macromolecule catabolic process;response to fungus;hydrolase activity, hydrolyzing O-glycosyl compounds;DNA metabolic process;cellular catabolic process;response to stress;anchored component of plasma membrane;single-stranded DNA binding;carbohydrate metabolic process;adenyl ribonucleotide binding;purine ribonucleoside triphosphate binding;cellular response to stimulus;integral component of membrane; |
| HORVU7Hr1G122100 | 5,58775194 | Pathogenesis protein 5 | response to biotic stimulus;ATP binding;glucan endo-1,3-beta-glucanase activity, C-3 substituted reducing group;glucan endo-1,4-beta-glucanase activity, C-3 substituted reducing group;response to external stimulus;integral component of membrane;killing of cells of other organism;protein kinase activity;defense response;extracellular region;protein phosphorylation; |
| HORVU3Hr1G096360 | 5,455179909 | Taxadienol acetyl transferase | transferase activity, transferring acyl groups other than amino-acyl groups;paclitaxel metabolic process;diterpenoid biosynthetic process; |
| HORVU2Hr1G002840 | 5,414319783 | L-type lectin-domain containing receptor kinase IX.1 | molecular transducer activity;response to salt stress;transmembrane receptor protein serine/threonine kinase signaling pathway;protein phosphorylation;response to water deprivation;ATP binding;response to cold;negative regulation of endopeptidase activity;peptidyl-tyrosine modification;regulation of cell cycle;carbohydrate binding;identical protein binding;zinc ion binding;2-alkenal reductase [NAD(P)] activity;protein kinase activity;regulation of apoptotic process;radial axis specification;embryonic meristem development;defense response to bacterium;plasma membrane;longitudinal axis specification;nucleic acid binding;peroxisome;abscisic acid-activated signaling pathway;transmembrane signaling receptor activity;cysteine-type endopeptidase inhibitor activity;defense response to oomycetes;integral component of membrane;oxidation-reduction process; |
| HORVU5Hr1G041660 | 5,344019314 | Putative LRR receptor-like serine/threonine-protein kinase | ATP binding;catalytic activity, acting on a protein;integral component of membrane;protein kinase activity;protein phosphorylation;phosphotransferase activity, alcohol group as acceptor;kinase activity; |
| HORVU7Hr1G057410 | 5,325469972 | MYB domain class transcription factor | nucleus;DNA binding;regulatory region nucleic acid binding;response to gibberellin;DNA-binding transcription factor activity;cell differentiation;integral component of membrane;regulation of transcription, DNA-templated;response to jasmonic acid;response to salicylic acid; |
| HORVU7Hr1G056820 | 5,30878819 | Heat-responsive transcription factor | negative regulation of biosynthetic process;double-stranded DNA binding;negative regulation of nitrogen compound metabolic process;sequence-specific DNA binding;regulation of phosphoprotein phosphatase activity;negative regulation of nucleic acid-templated transcription;RNA polymerase II regulatory region DNA binding;negative regulation of hydrolase activity;DNA-binding transcription factor activity;cellular response to heat;negative regulation of cellular macromolecule biosynthetic process;zinc ion binding;serine-type endopeptidase activity;regulation of transcription, DNA-templated;asymmetric cell division;regulation of signal transduction;nucleus;negative regulation of gene expression;protein phosphatase inhibitor activity;positive regulation of nucleic acid-templated transcription;cytoplasm;positive regulation of gene expression;proteolysis;negative regulation of macromolecule metabolic process;integral component of membrane;response to chitin;negative regulation of cellular metabolic process; |
| HORVU3Hr1G001020 | 5,301719735 | Wall-associated receptor kinase 3 | ATP binding;calcium ion binding;integral component of membrane;protein kinase activity;protein phosphorylation;polysaccharide binding; |
| HORVU2Hr1G000590 | 5,253051557 | Disease resistance protein RGA2 | ADP binding; |
| HORVU5Hr1G122390 | 5,208574338 | Putative LRR receptor-like serine/threonine-protein kinase | ATP binding;integral component of membrane;protein kinase activity;peptidyl-tyrosine modification;protein phosphorylation; |
| HORVU3Hr1G032410 | 5,180955999 | Putative transcription factor OsGLK2 (Fragment) | nucleus;DNA binding; |
| HORVU3Hr1G002270 | 5,173819916 | Receptor kinase LRK10 | ATP binding;integral component of membrane;protein kinase activity;protein phosphorylation;plasma membrane; |
| HORVU1Hr1G092310 | 5,172966987 | Glucan endo-1,3-beta-glucosidase 13 | carbohydrate metabolic process;hydrolase activity, hydrolyzing O-glycosyl compounds; |
| HORVU4Hr1G076420 | 5,172679427 | 'putative 1,4-benzoquinone reductase | NAD(P)H dehydrogenase (quinone) activity;FMN binding;oxidation-reduction process; |
| HORVU2Hr1G001380 | 5,164882869 | Peptide transporter PTR2 | oligopeptide transport;transmembrane transport;transmembrane transporter activity;integral component of membrane; |
| HORVU7Hr1G089480 | 5,122165503 | Peroxidase | extracellular region;plant-type cell wall;hydrogen peroxide catabolic process;heme binding;peroxidase activity;response to oxidative stress;defense response to bacterium, incompatible interaction;plasmodesma;metal ion binding;cytoplasm;cellular oxidant detoxification;integral component of membrane;oxidation-reduction process; |
| HORVU6Hr1G050970 | 5,113518486 | Peptidyl-prolyl cis-trans isomerase | cyclosporin A binding;unfolded protein binding;protein peptidyl-prolyl isomerization;protein folding;extracellular region;root development;peptidyl-prolyl cis-trans isomerase activity;chloroplast;plasmodesma;membrane;endomembrane system;cytoplasmic vesicle;cytosol;regulation of protein refolding;organelle subcompartment; |
| HORVU2Hr1G002310 | 5,062085716 | Zinc finger, CCHC-type | nucleic acid binding;ATP binding;DNA repair;carbohydrate metabolic process;zinc ion binding;DNA helicase activity;DNA duplex unwinding;DNA integration;DNA recombination;telomere maintenance;hydrolase activity, hydrolyzing O-glycosyl compounds; |
|  |  |  |  |
| HORVU1Hr1G024180 | -5,003031462 | Arabinogalactan protein 2 | somatic embryogenesis;response to salt stress;cell morphogenesis involved in differentiation;seed trichome differentiation;double-stranded DNA binding;regulation of transcription, DNA-templated;cell adhesion;plant-type secondary cell wall biogenesis;response to cytokinin;plasma membrane;response to auxin;response to gibberellin;unidimensional cell growth;response to abscisic acid;regulation of protein localization to cell surface;intrinsic component of membrane; |
| HORVU0Hr1G005360 | -5,029051026 | Carboxypeptidase | secondary metabolic process;proteolysis;serine-type carboxypeptidase activity; |
| HORVU2Hr1G126130 | -5,030800409 | Phenol hydroxylase reductase | oxidoreductase activity;oxidation-reduction process; |
| HORVU7Hr1G036890 | -5,080337238 | Glycosyltransferase | UDP-glycosyltransferase activity;transferase activity, transferring hexosyl groups;intracellular membrane-bounded organelle;integral component of membrane; |
| HORVU2Hr1G119220 | -5,110794806 | WD repeat-containing protein 44 | 0 |
| HORVU5Hr1G034100 | -5,115770125 | Fasciclin-like arabinogalactan-protein-like | integral component of membrane;cell adhesion;vacuolar membrane;anchored component of plasma membrane; |
| HORVU2Hr1G089620 | -5,124280847 | Cytokinin riboside 5'-monophosphate phosphoribohydrolase | cytokinin biosynthetic process;nucleus;lyase activity;cytosol;hydrolase activity; |
| HORVU1Hr1G088920 | -5,135005925 | Chlorophyll a-b binding protein, chloroplastic | photosynthesis, light harvesting;photosystem II;chloroplast envelope;photosystem I;regulation of transcription, DNA-templated;response to light stimulus;chloroplast thylakoid membrane;nucleic acid binding;DNA binding;DNA-binding transcription factor activity;metal ion binding;integral component of membrane;protein-chromophore linkage;chlorophyll binding;plastoglobule; |
| HORVU2Hr1G076010 | -5,146431016 | Photosystem II oxygen-evolving enhancer protein 2 | plastid thylakoid lumen;chloroplast;transmembrane transport;transmembrane transporter activity;calcium ion binding;integral component of membrane;photosystem II oxygen evolving complex;photosynthesis;extrinsic component of membrane;hydrolase activity, hydrolyzing O-glycosyl compounds;NAD+ binding; |
| HORVU1Hr1G079290 | -5,152628992 | Late embryogenesis abundant protein 3 | nucleus;positive regulation of response to water deprivation;integral component of membrane;bacteriocin transport;toxin transport;toxin transmembrane transporter activity; |
| HORVU7Hr1G030180 | -5,164325674 | Salt stress-induced protein | nucleus;DNA binding;zinc ion binding;integral component of membrane;regulation of transcription, DNA-templated;carbohydrate binding; |
| HORVU7Hr1G036720 | -5,185822091 | Germin-like protein 8-14 | molybdenum ion binding;anion binding;nutrient reservoir activity;coenzyme binding;oxidoreductase activity;apoplast;cell wall;integral component of membrane;ribonucleotide binding;oxidation-reduction process;manganese ion binding;extracellular matrix;vitamin B6 binding; |
| HORVU6Hr1G090200 | -5,208215166 | Phosphoribosyltransferase | integral component of membrane; |
| HORVU1Hr1G002090 | -5,224140797 | Arginine decarboxylase | arginine catabolic process;response to cold;arginine decarboxylase activity;metal ion binding;spermidine biosynthetic process;putrescine biosynthetic process;oxidation-reduction process;monooxygenase activity; |
| HORVU0Hr1G022690 | -5,233970106 | Senescence-associated protein DIN1 | response to oxidative stress;chloroplast;protein binding;response to wounding;integral component of membrane;response to jasmonic acid;thiosulfate sulfurtransferase activity;aging;thylakoid; |
| HORVU2Hr1G099370 | -5,247221088 | Bifunctional inhibitor/lipid-transfer protein/seed storage 2S albumin superfamily protein isoform 1 | GTPase activator activity;DNA integration;actin filament organization;structural constituent of cell wall;extracellular region;protein phosphorylation;positive regulation of GTPase activity;chloroplast thylakoid membrane;GTP binding;ATP binding;plasmodesma;GTPase activity;Rac GTPase binding;cytosol;potassium ion transmembrane transport;signal transduction;DNA recombination;protein serine/threonine kinase activity;nucleic acid binding;nucleus;calmodulin binding;regulation of actin cytoskeleton organization;cell wall;integral component of membrane;small conductance calcium-activated potassium channel activity;calcium-ion regulated exocytosis; |
| HORVU1Hr1G000340 | -5,284629711 | Gene encoding jasmonate-induced protein (Fragment) | 0 |
| HORVU2Hr1G107670 | -5,288511492 | 0 | carbohydrate metabolic process;cellular macromolecule metabolic process;macromolecule catabolic process;cell communication;integral component of membrane;hydrolase activity, hydrolyzing O-glycosyl compounds;carbohydrate binding;transferase activity;cellular catabolic process; |
| HORVU5Hr1G112670 | -5,305939361 | Chalcone--flavonone isomerase | DNA binding;nucleus;extrinsic component of endoplasmic reticulum membrane;plant-type vacuole membrane;pigmentation;response to karrikin;regulation of transcription, DNA-templated;intramolecular lyase activity;flavonoid biosynthetic process;response to UV-B;response to auxin; |
| HORVU2Hr1G080350 | -5,315818567 | Amino acid/polyamine transporter II | integral component of membrane;amino acid transmembrane transporter activity;amino acid transmembrane transport;plasma membrane; |
| HORVU7Hr1G049860 | -5,345416058 | LOW QUALITY PROTEIN: fasciclin-like arabinogalactan protein 7 | seed trichome differentiation;cell adhesion;steryl-sulfatase activity;plant-type secondary cell wall biogenesis;response to cytokinin;response to stress;anchored component of plasma membrane;response to auxin;actin filament binding;response to gibberellin;response to abiotic stimulus;response to abscisic acid;integral component of membrane;actin filament bundle assembly; |
| HORVU3Hr1G070210 | -5,349179794 | Aminotransferase, class I/classII | pyridoxal phosphate binding;biotin metabolic process;catalytic activity;integral component of membrane;cytosol;biosynthetic process;microbody; |
| HORVU3Hr1G099550 | -5,411766394 | Eukaryotic aspartyl protease family protein | xylan catabolic process;aspartic-type endopeptidase activity;proteolysis; |
| HORVU4Hr1G061740 | -5,45123603 | Phospholipase A2-alpha | phospholipase A2 activity (consuming 1,2-dipalmitoylphosphatidylcholine);phospholipase A2 activity consuming 1,2-dioleoylphosphatidylethanolamine);pollen development;endoplasmic reticulum;lipid binding;arachidonate transport;extracellular region;phospholipid metabolic process;response to light stimulus;phospholipase A2 activity;icosanoid secretion;regulation of stomatal movement;calcium ion binding;lipid catabolic process;integral component of membrane; |
| HORVU0Hr1G038220 | -5,454787617 | High molecular mass early light-inducible protein HV58, chloroplastic | positive regulation of seed germination;regulation of chlorophyll biosynthetic process;response to karrikin;photosystem II;photosystem I;cellular response to far red light;cellular response to blue light;response to cold;chloroplast;cellular response to red light;cellular response to UV-A;cellular response to heat;cellular response to high light intensity;integral component of membrane;plastid thylakoid membrane;photosynthesis;photoprotection; |
| HORVU3Hr1G039220 | -5,455278537 | E3 ubiquitin-protein ligase RING1 | ubiquitin protein ligase activity;transferase activity, transferring acyl groups;intracellular organelle part;ubiquitin-dependent protein catabolic process;cytoplasm;metal ion binding;bounding membrane of organelle;intracellular membrane-bounded organelle;integral component of membrane;response to chitin;ligase activity;whole membrane;protein ubiquitination; |
| HORVU7Hr1G043690 | -5,52480312 | UDP-glucose:2-hydroxyflavanone C-glucosyltransferase | UDP-glycosyltransferase activity;glycine-tRNA ligase activity;ATP binding;glycyl-tRNA aminoacylation;transferase activity, transferring hexosyl groups;cytoplasm;intracellular membrane-bounded organelle;integral component of membrane; |
| HORVU6Hr1G068370 | -5,52631696 | Growth-regulating factor | cell population proliferation;transcription, DNA-templated;negative regulation of cell population proliferation;transcription regulatory region DNA binding;regulation of transcription, DNA-templated;reproductive process;sequence-specific DNA binding;response to nematode;nucleus;ATP binding;positive regulation of nucleic acid-templated transcription;protein binding;positive regulation of gene expression;regulation of nematode larval development;developmental process;multicellular organismal process; |
| HORVU4Hr1G066800 | -5,527251165 | High molecular mass early light-inducible protein HV58, chloroplastic | positive regulation of seed germination;regulation of chlorophyll biosynthetic process;photosystem II;response to karrikin;photosystem I;thylakoid membrane;cellular response to far red light;plastid part;organelle membrane;organelle envelope;cellular response to blue light;response to cold;chloroplast;cellular response to red light;cellular response to UV-A;cellular response to heat;cellular response to high light intensity;integral component of membrane;photosynthesis;photoprotection;response to UV-B;organelle subcompartment; |
| HORVU5Hr1G119480 | -5,541896486 | Ice recrystallization inhibition protein IRI | ATP binding;catalytic activity, acting on a protein;integral component of membrane;cellular protein modification process;phosphorylation;phosphotransferase activity, alcohol group as acceptor;kinase activity; |
| HORVU4Hr1G028720 | -5,548129231 | Xyloglucan endotransglucosylase/hydrolase | hydrolase activity, hydrolyzing O-glycosyl compounds;cell wall biogenesis;response to low light intensity stimulus;response to mechanical stimulus;response to auxin;chloroplast;unidimensional cell growth;plasmodesma;metal ion binding;apoplast;cell wall;membrane;cell wall organization;metal ion transport;xyloglucan:xyloglucosyl transferase activity;carbohydrate binding;xyloglucan metabolic process; |
| HORVU0Hr1G002100 | -5,554633901 | ATP-dependent DNA helicase | DNA repair;DNA replication;DNA recombination;four-way junction helicase activity;chromosome;SOS response;3'-5' DNA helicase activity;nucleic acid binding;nucleus;ATP binding;cytoplasm;DNA duplex unwinding;integral component of membrane; |
| HORVU5Hr1G000620 | -5,56732582 | High molecular mass early light-inducible protein HV58, chloroplastic | positive regulation of seed germination;regulation of chlorophyll biosynthetic process;response to karrikin;photosystem II;photosystem I;cellular response to far red light;cellular response to blue light;response to cold;chloroplast;cellular response to red light;cellular response to UV-A;cellular response to heat;cellular response to high light intensity;integral component of membrane;plastid thylakoid membrane;photosynthesis;photoprotection; |
| HORVU2Hr1G104030 | -5,58862665 | Concanavalin A-like lectin/glucanase, subgroup | defense response by callose deposition in cell wall;ATP binding;identical protein binding;endosome;integral component of membrane;protein kinase activity;detection of bacterium;regulation of anion channel activity;protein phosphorylation;defense response to bacterium;receptor-mediated endocytosis;plasma membrane; |
| HORVU7Hr1G098440 | -5,596525298 | Xyloglucan endotransglucosylase/hydrolase | apoplast;cell wall;integral component of membrane;cell wall organization;xyloglucan:xyloglucosyl transferase activity;hydrolase activity, hydrolyzing O-glycosyl compounds;carbohydrate binding;xyloglucan metabolic process;cell wall biogenesis; |
| HORVU4Hr1G044140 | -5,631012956 | Monogalactosyldiacylglycerol synthase 2, chloroplastic (Fragment) | UDP-glycosyltransferase activity;transferase activity, transferring hexosyl groups;response to salt stress;glycolipid biosynthetic process;glycerolipid biosynthetic process;plastid outer membrane;response to cytokinin;response to light stimulus;fatty acid metabolic process;Arp2/3 protein complex;chloroplast thylakoid membrane;response to water deprivation;response to gibberellin;response to cold;response to deep water;chloroplast membrane;galactolipid metabolic process;Arp2/3 complex-mediated actin nucleation;integral component of membrane;cellular response to phosphate starvation; |
| HORVU7Hr1G102030 | -5,642957324 | Bifunctional inhibitor/plant lipid transfer protein/seed storage helical domain | lipid transport;systemic acquired resistance;fatty acid binding;proteolysis;integral component of membrane;Rho guanyl-nucleotide exchange factor activity;peptidase activity; |
| HORVU5Hr1G113060 | -5,646947683 | Actin depolymerizing factor | contractile ring;zinc ion binding;medial cortex;actin filament depolymerization;mitotic actomyosin contractile ring assembly;Golgi to plasma membrane protein transport;defense response;endocytosis;negative regulation of actin binding;regulation of actin filament binding;endocytic patch;plasma membrane;protein-containing complex binding;nucleic acid binding;nucleus;actin filament severing;actin binding;cytoskeletal part;actin nucleation;actin cytoskeleton;integral component of membrane;cortical cytoskeleton; |
| HORVU5Hr1G066760 | -5,687767392 | Pentatricopeptide repeat-containing protein | integral component of membrane; |
| HORVU3Hr1G078250 | -5,690017572 | Methyladenine glycosylase family protein | base-excision repair;DNA-3-methylbase glycosylase activity; |
| HORVU7Hr1G021120 | -5,697059365 | phosphatidylinositol transfer protein 3-like isoform X2 | 0 |
| HORVU5Hr1G080350 | -5,71398799 | C-repeat binding factor | nucleus;DNA binding;DNA-binding transcription factor activity;peroxisome fission;integral component of peroxisomal membrane;regulation of transcription, DNA-templated; |
| HORVU1Hr1G087740 | -5,718435739 | Early light-inducible protein ELIP | organelle envelope;chloroplast;integral component of membrane;thylakoid membrane;plastid part;organelle membrane;organelle subcompartment; |
| HORVU3Hr1G089910 | -5,726745803 | Trigger factor, ribosome-binding | protein transport;protein folding; |
| HORVU1Hr1G069380 | -5,782305289 | KH domain-containing protein | nucleus;DNA binding;RNA binding;protein binding;negative regulation of short-day photoperiodism, flowering;flower development;integral component of membrane;negative regulation of long-day photoperiodism, flowering; |
| HORVU1Hr1G088900 | -5,815293173 | Chlorophyll a-b binding protein, chloroplastic | metal ion binding;photosynthesis, light harvesting;photosystem II;integral component of membrane;chloroplast envelope;photosystem I;protein-chromophore linkage;chlorophyll binding;response to light stimulus;plastoglobule;chloroplast thylakoid membrane; |
| HORVU4Hr1G062070 | -5,882238578 | Solute carrier family 40 protein | cellular iron ion homeostasis;iron ion transmembrane transporter activity;integral component of membrane;chloroplast envelope;iron ion transmembrane transport; |
| HORVU4Hr1G086020 | -5,910980977 | Dihydrodipicolinate reductase | chloroplast stroma;structural molecule activity;proteasome assembly;intracellular protein transport;RNA metabolic process;plasmodesma;vesicle-mediated transport;4-hydroxy-tetrahydrodipicolinate reductase;cytosol;photosynthesis, light reaction;ribonucleoprotein complex;cellular macromolecule biosynthetic process;lysine biosynthetic process;zinc ion binding;dicarboxylic acid biosynthetic process;proteasome complex;response to cadmium ion;ubiquitin-dependent protein catabolic process;nucleic acid binding;nucleus;DNA-directed 5'-3' RNA polymerase activity;diaminopimelate metabolic process;integral component of membrane;gene expression;nucleobase-containing compound biosynthetic process;oxidation-reduction process; |
| HORVU2Hr1G112120 | -5,941978052 | 0 | transferase activity, transferring acyl groups other than amino-acyl groups; |
| HORVU0Hr1G019300 | -5,958827428 | Glutathione S-transferase, N-terminal domain containing protein, expressed | cytoplasm;glutathione metabolic process;glutathione transferase activity; |
| HORVU6Hr1G012270 | -5,99066097 | Putative terpene synthase | magnesium ion binding;chloroplast;monoterpene biosynthetic process;defense response to bacterium;terpenoid biosynthetic process;terpene synthase activity;S-linalool synthase activity; |
| HORVU0Hr1G026250 | -6,009295883 | Ribosomal protein L14 | chloroplast;structural constituent of ribosome;ribosome binding;mitochondrial ribosome;ribosome assembly;large ribosomal subunit;rRNA binding;plastid ribosome;translation; |
| HORVU2Hr1G000080 | -6,015121043 | probable protein phosphatase 2C 75 | cation binding;protein phosphorylation;negative regulation of protein kinase activity;response to water deprivation;hormone-mediated signaling pathway;ATP binding;response to cold;response to heat;regulation of stomatal movement;cellular response to lipid;cellular response to alcohol;cytosol;protein kinase binding;positive regulation of seed germination;protein dephosphorylation;glycolytic process;response to high light intensity;response to osmotic stress;negative regulation of photosynthesis, light reaction;protein kinase activity;plasma membrane;negative regulation of abscisic acid-activated signaling pathway;protein serine/threonine phosphatase activity;nucleus;pyruvate kinase activity;response to abscisic acid;metal ion binding;integral component of membrane;negative regulation of transforming growth factor beta receptor signaling pathway;cellular response to acid chemical; |
| HORVU3Hr1G003540 | -6,031706207 | Glycoside hydrolase | carbohydrate metabolic process;cell wall macromolecule catabolic process;chloroplast;plasmodesma;apoplast;integral component of membrane;cytosol;response to cadmium ion;cell wall organization;carbohydrate binding;plant-type cell wall;hydrolase activity, hydrolyzing O-glycosyl compounds; |
| HORVU7Hr1G058120 | -6,03397618 | Chlorophyll a-b binding protein, chloroplastic | photosynthesis, light harvesting;photosystem II;chloroplast envelope;photosystem I;regulation of transcription, DNA-templated;response to light stimulus;chloroplast thylakoid membrane;nucleic acid binding;carbohydrate metabolic process;DNA-binding transcription factor activity;metal ion binding;catalytic activity;integral component of membrane;protein-chromophore linkage;chlorophyll binding;carbohydrate binding;plastoglobule; |
| HORVU7Hr1G021000 | -6,114792417 | BAHD acyltransferase DCR | transferase activity, transferring acyl groups other than amino-acyl groups;transmembrane transport;metal ion binding;proteolysis;carbohydrate transport;integral component of membrane;metalloendopeptidase activity; |
| HORVU5Hr1G046480 | -6,118419339 | Chalcone-flavonone isomerase family protein (Fragment) | calcium ion binding;response to karrikin;integral component of membrane;intramolecular lyase activity; |
| HORVU7Hr1G087270 | -6,119002603 | MACPF domain-containing protein isoform A | plant-type hypersensitive response;plasma membrane; |
| HORVU4Hr1G063190 | -6,138656982 | Early light-inducible protein ELIP | positive regulation of seed germination;regulation of chlorophyll biosynthetic process;photosystem II;response to karrikin;photosystem I;cellular response to far red light;cellular response to blue light;response to cold;chloroplast;cellular response to red light;cellular response to UV-A;cellular response to heat;cellular response to high light intensity;integral component of membrane;plastid thylakoid membrane;photosynthesis;photoprotection;response to UV-B; |
| HORVU0Hr1G013210 | -6,140167973 | 3-methyl-2-oxobutanoate hydroxymethyltransferase (Fragment) | heat shock protein binding;vesicle-mediated transport;metal ion binding;integral component of membrane;clathrin adaptor complex;unfolded protein binding;methyltransferase activity;intracellular protein transport;methylation; |
| HORVU5Hr1G071940 | -6,161369074 | Cyanohydrin beta-glucosyltransferase | UDP-glycosyltransferase activity;dhurrin biosynthetic process;transferase activity, transferring hexosyl groups;tRNA (guanine-N7-)-methyltransferase activity;glycolytic process;cytoplasmic part;membrane part;organelle membrane;intracellular organelle part;intracellular membrane-bounded organelle;phosphoglycerate kinase activity;nuclear outer membrane-endoplasmic reticulum membrane network;tRNA (guanine-N7)-methylation; |
| HORVU6Hr1G091650 | -6,247548969 | Chlorophyll a-b binding protein, chloroplastic | photosynthesis, light harvesting;photosystem II;chloroplast envelope;photosystem I;regulation of transcription, DNA-templated;response to light stimulus;chloroplast thylakoid membrane;DNA binding;DNA-binding transcription factor activity;metal ion binding;integral component of membrane;protein-chromophore linkage;chlorophyll binding;plastoglobule; |
| HORVU6Hr1G091660 | -6,247548969 | Chlorophyll a-b binding protein, chloroplastic | photosynthesis, light harvesting;photosystem II;chloroplast envelope;photosystem I;regulation of transcription, DNA-templated;response to light stimulus;chloroplast thylakoid membrane;DNA binding;DNA-binding transcription factor activity;metal ion binding;integral component of membrane;protein-chromophore linkage;chlorophyll binding;plastoglobule; |
| HORVU2Hr1G072880 | -6,279870942 | Ferredoxin | chloroplast;metal ion binding;electron transfer activity;cyanelle;integral component of membrane;electron transport chain;ribosome;2 iron, 2 sulfur cluster binding; |
| HORVU7Hr1G046430 | -6,310233745 | Rhoptry neck protein 2 (Fragment) | DNA repair;determination of adult lifespan;protein transport;snoRNA processing;helicase activity;Golgi membrane;RNA splicing, via transesterification reactions with bulged adenosine as nucleophile;polytene chromosome;5'-3' exoribonuclease activity;exoribonuclease II activity;RNA binding;positive regulation of transcription, DNA-templated;nematode larval development;protein localization to peroxisome;cytosol;nuclear-transcribed mRNA catabolic process, exonucleolytic, 5'-3';negative regulation of protein metabolic process;RNA phosphodiester bond hydrolysis, exonucleolytic;zinc ion binding;positive regulation of DNA topoisomerase (ATP-hydrolyzing) activity;retrograde vesicle-mediated transport, Golgi to endoplasmic reticulum;protein-DNA complex assembly;thiamine metabolic process;chromatin;hyperosmotic response;nucleus;regulation of mRNA processing;peroxisomal membrane transport;protein ubiquitination;DNA replication;extracellular region;ATP binding;termination of RNA polymerase II transcription, poly(A)-coupled;response to heat;pseudouridine synthesis;dosage compensation;nuclear polyadenylation-dependent rRNA catabolic process;termination of RNA polymerase II transcription, exosome-dependent;DNA duplex unwinding;positive regulation of heterochromatin assembly;regulation of cellular amide metabolic process;negative regulation of DNA-templated transcription, elongation;regulation of cellular protein metabolic process;phosphorylation;COPI vesicle coat;cytoplasmic ribonucleoprotein granule;thiamine-containing compound biosynthetic process;DNA-dependent ATPase activity;catalytic activity, acting on DNA;transmembrane transport;X chromosome;germ plasm;DNA recombination;phosphomethylpyrimidine kinase activity;cleavage in ITS2 between 5.8S rRNA and LSU-rRNA of tricistronic rRNA transcript (SSU-rRNA, 5.8S rRNA, LSU-rRNA);male courtship behavior, veined wing generated song production;chromatin binding;pseudouridine synthase activity;mRNA processing;regulation of transcription by RNA polymerase II;negative regulation of phosphorylation;posttranscriptional regulation of gene expression;establishment of protein localization to organelle;peroxisome;regulatory region nucleic acid binding;DNA binding;RNA-DNA hybrid ribonuclease activity;ubiquitin-protein transferase activity;axon extension;intracellular organelle lumen;lipid metabolic process;integral component of membrane;nuclear mRNA surveillance;protein acetyltransferase complex; |
| HORVU1Hr1G053950 | -6,357712084 | PolyADP-ribose polymerase | nucleic acid binding;integral component of membrane; |
| HORVU2Hr1G010690 | -6,390333208 | Ribulose bisphosphate carboxylase small chain | DNA-dependent DNA replication;photorespiration;monooxygenase activity;plastid;DNA binding;ATP binding;DNA-directed DNA polymerase activity;carbohydrate biosynthetic process;DNA biosynthetic process;photosynthesis;oxidation-reduction process;carbon fixation;ribulose-bisphosphate carboxylase activity; |
| HORVU0Hr1G016640 | -6,420158127 | P-loop containing nucleoside triphosphate hydrolase | ATP binding;zinc ion binding;mitochondrion;mitochondrion organization;hydrolase activity; |
| HORVU6Hr1G014270 | -6,442200511 | Plant-specific FAD-dependent oxidoreductase | FAD binding;eukaryotic 48S preinitiation complex;eukaryotic translation initiation factor 3 complex;D-arabinono-1,4-lactone oxidase activity;translation initiation factor activity;formation of cytoplasmic translation initiation complex;eukaryotic 43S preinitiation complex;membrane;cell wall;oxidation-reduction process;L-gulonolactone oxidase activity;translation initiation factor binding;L-ascorbic acid biosynthetic process;galactonolactone dehydrogenase activity; |
| HORVU4Hr1G009800 | -6,454364418 | Amino cyclopropane carboxylate acid synthase | 1-aminocyclopropane-1-carboxylate biosynthetic process;pyridoxal phosphate binding;phloem or xylem histogenesis;1-aminocyclopropane-1-carboxylate synthase activity;transaminase activity;ethylene metabolic process;fruit ripening;cellular response to iron ion;response to cytokinin;hormone biosynthetic process;regulation of translation;cell division;protein binding;oxidoreductase activity;alkene biosynthetic process;oxidation-reduction process; |
| HORVU1Hr1G056280 | -6,478172879 | GATA transcription factor | nucleus;zinc ion binding;DNA-binding transcription factor activity;positive regulation of transcription, DNA-templated;sequence-specific DNA binding;response to light stimulus; |
| HORVU6Hr1G066420 | -6,499053931 | Spermidine hydroxycinnamoyl transferase | transferase activity, transferring acyl groups other than amino-acyl groups;integral component of membrane; |
| HORVU3Hr1G081180 | -6,513332501 | Expansin | unidimensional cell growth;structural constituent of ribosome;primary root development;cell wall;membrane;plant-type cell wall organization;extracellular region;ribosome;translation; |
| HORVU6Hr1G083960 | -6,526368373 | Dehydrin | response to abscisic acid;cytosol;cold acclimation;response to water; |
| HORVU3Hr1G117770 | -6,560338354 | 0 | regulation of transcription, DNA-templated;intracellular part;translational elongation;membrane-bounded organelle;response to cold;DNA binding;DNA-binding transcription factor activity;protein binding;translation elongation factor activity;metal ion binding;integral component of membrane;response to chitin;defense response to fungus; |
| HORVU5Hr1G107020 | -6,565663572 | Eukaryotic translation initiation factor 2c, putative | zinc ion binding;female meiotic nuclear division;megagametogenesis;male meiotic nuclear division;regulation of transcription, DNA-templated;translational initiation;nucleolus organizer region;nucleic acid binding;translation regulator activity;cytoplasm;microgametogenesis;integral component of membrane;gene silencing by RNA;histone H3-K9 demethylation; |
| HORVU3Hr1G079900 | -6,566981425 | Glycerophosphoryl diester phosphodiesterase | lipid metabolic process;integral component of membrane;organophosphate catabolic process;phosphoric diester hydrolase activity;cellular catabolic process;phosphate-containing compound metabolic process; |
| HORVU2Hr1G099820 | -6,590308811 | Cold-responsive protein Wcs19-B | lipid transport;response to heat;iron ion binding;electron transfer activity;integral component of membrane;response to desiccation;lipid binding;lipoprotein metabolic process;electron transport chain;extracellular region;hyperosmotic response;heme binding; |
| HORVU5Hr1G063620 | -6,595871168 | Low molecular mass early light-inducible protein HV90, chloroplastic | organelle envelope;chloroplast;integral component of membrane;thylakoid membrane;plastid part;organelle membrane;organelle subcompartment; |
| HORVU1Hr1G078290 | -6,630660743 | 0 | chloroplast stroma;lysine biosynthetic process;diaminopimelate metabolic process;4-hydroxy-tetrahydrodipicolinate reductase;integral component of membrane;photosynthesis, light reaction;oxidation-reduction process; |
| HORVU2Hr1G113850 | -6,637907767 | 0 | U2AF;multicellular organism development;positive regulation of cell population proliferation;transcription by RNA polymerase II;helicase activity;extracellular region;RNA splicing, via transesterification reactions with bulged adenosine as nucleophile;ATP binding;RNA binding;transcription factor TFIID complex;cell wall organization;catalytic activity, acting on RNA;exonuclease activity, active with either ribo- or deoxyribonucleic acids and producing 5'-phosphomonoesters;mRNA transport;tRNA-splicing ligase complex;RNA phosphodiester bond hydrolysis, exonucleolytic;chitosanase activity;protein methyltransferase activity;DNA-templated transcription, initiation;mRNA processing;positive regulation of ruffle assembly;protein methylation;structural constituent of cuticle;lysosome localization;5'-3' exonuclease activity;RNA cap binding complex;polysaccharide catabolic process;metal ion binding;positive regulation of gene expression;cell wall;integral component of membrane;RNA 5'-cap (guanine-N7)-methylation; |
| HORVU2Hr1G095080 | -6,642820925 | Cytochrome P450 family protein, expressed | taxane 13-alpha-hydroxylase activity;iron ion binding;oxidation-reduction process;heme binding; |
| HORVU0Hr1G019640 | -6,685309855 | Myc-type, basic helix-loop-helix (BHLH) domain-containing protein | nucleus;regulation of short-day photoperiodism, flowering;regulation of long-day photoperiodism, flowering;integral component of membrane;protein dimerization activity;DNA-binding transcription activator activity, RNA polymerase II-specific;positive regulation of transcription by RNA polymerase II;hydrolase activity; |
| HORVU2Hr1G010690 | -6,704670227 | Ribulose bisphosphate carboxylase small chain | carbohydrate biosynthetic process;photorespiration;photosynthesis;oxidation-reduction process;carbon fixation;monooxygenase activity;ribulose-bisphosphate carboxylase activity;plastid; |
| HORVU2Hr1G073210 | -6,739558883 | 0 | carbohydrate metabolic process;carbohydrate localization;cell wall;apoplast;integral component of membrane;hydrolase activity, hydrolyzing O-glycosyl compounds;defense response to fungus;defense response to bacterium; |
| HORVU4Hr1G074740 | -6,796357919 | Major facilitator superfamily domain-containing protein 12 | transmembrane transport;carbohydrate transport;integral component of membrane;symporter activity;intrinsic component of plasma membrane; |
| HORVU3Hr1G078260 | -6,839982381 | Putative Glutamine amidotransferase | base-excision repair;phosphorylation;kinase activity;DNA-3-methylbase glycosylase activity; |
| HORVU4Hr1G066810 | -6,875258945 | High molecular mass early light-inducible protein HV58, chloroplastic | positive regulation of seed germination;regulation of chlorophyll biosynthetic process;photosystem II;response to karrikin;photosystem I;thylakoid membrane;cellular response to far red light;plastid part;organelle membrane;organelle envelope;cellular response to blue light;response to cold;chloroplast;cellular response to red light;cellular response to UV-A;cellular response to heat;cellular response to high light intensity;integral component of membrane;photosynthesis;photoprotection;response to UV-B;organelle subcompartment; |
| HORVU5Hr1G077390 | -6,907716909 | BHLH transcription factor | nucleus;ATP binding;DNA-binding transcription factor activity;transcription, DNA-templated;integral component of membrane;regulation of transcription, DNA-templated;protein dimerization activity;sequence-specific DNA binding;protein kinase activity;protein phosphorylation;positive regulation of shade avoidance;polysaccharide binding; |
| HORVU1Hr1G067080 | -7,132281044 | High molecular mass early light-inducible protein HV58, chloroplastic | positive regulation of seed germination;regulation of chlorophyll biosynthetic process;photosystem II;response to karrikin;photosystem I;cellular response to far red light;cellular response to blue light;response to cold;chloroplast;cellular response to red light;cellular response to UV-A;cellular response to heat;cellular response to high light intensity;integral component of membrane;plastid thylakoid membrane;photosynthesis;photoprotection;response to UV-B; |
| HORVU5Hr1G080440 | -7,134496755 | C-repeat binding factor roughrider | nucleus;DNA binding;DNA-binding transcription factor activity;regulation of transcription, DNA-templated; |
| HORVU0Hr1G023360 | -7,188061903 | High molecular mass early light-inducible protein HV58, chloroplastic | positive regulation of seed germination;regulation of chlorophyll biosynthetic process;response to karrikin;photosystem II;photosystem I;cellular response to far red light;cellular response to blue light;response to cold;chloroplast;cellular response to red light;cellular response to UV-A;cellular response to heat;cellular response to high light intensity;integral component of membrane;plastid thylakoid membrane;photosynthesis;photoprotection; |
| HORVU2Hr1G010690 | -7,189694302 | Ribulose bisphosphate carboxylase small chain | carbohydrate biosynthetic process;photorespiration;photosynthesis;oxidation-reduction process;carbon fixation;monooxygenase activity;ribulose-bisphosphate carboxylase activity;plastid; |
| HORVU1Hr1G049190 | -7,259766284 | Glutathione-S-transferase 2 | cytoplasm;glutathione metabolic process;glutathione transferase activity; |
| HORVU3Hr1G075210 | -7,281022547 | Expansin | unidimensional cell growth;structural constituent of ribosome;primary root development;cell wall;membrane;plant-type cell wall organization;extracellular region;ribosome;translation; |
| HORVU0Hr1G008960 | -7,460477109 | High molecular mass early light-inducible protein HV58, chloroplastic | positive regulation of seed germination;regulation of chlorophyll biosynthetic process;photosystem II;response to karrikin;photosystem I;thylakoid membrane;cellular response to far red light;plastid part;organelle membrane;organelle envelope;cellular response to blue light;response to cold;chloroplast;cellular response to red light;cellular response to UV-A;cellular response to heat;cellular response to high light intensity;integral component of membrane;photosynthesis;photoprotection;response to UV-B;organelle subcompartment; |
| HORVU6Hr1G056160 | -7,521920169 | Putative methyltransferase PMT13 | trans-Golgi network;endosome;integral component of membrane;methyltransferase activity;Golgi membrane;cell wall pectin metabolic process;methylation; |
| HORVU7Hr1G040380 | -7,545068952 | Chlorophyll a-b binding protein, chloroplastic | nucleic acid binding;metal ion binding;photosynthesis, light harvesting;photosystem II;integral component of membrane;chloroplast envelope;photosystem I;protein-chromophore linkage;chlorophyll binding;response to light stimulus;plastoglobule;chloroplast thylakoid membrane; |
| HORVU3Hr1G073180 | -7,585531059 | Solute carrier family 23 member 2 | transmembrane transport;transmembrane transporter activity;integral component of membrane; |
| HORVU0Hr1G030110 | -7,731967273 | 0 | nucleus;zinc ion binding;regulation of flower development;circadian rhythm;protein binding, bridging; |
| HORVU5Hr1G063570 | -7,823159648 | Low molecular mass early light-inducible protein HV90, chloroplastic | organelle envelope;chloroplast;integral component of membrane;thylakoid membrane;plastid part;organelle membrane;organelle subcompartment; |
| HORVU1Hr1G000440 | -8,052623295 | Jasmonate induced protein | integral component of membrane; |
| HORVU2Hr1G010670 | -8,182577688 | Ribulose bisphosphate carboxylase small chain | carbohydrate biosynthetic process;photorespiration;photosynthesis;oxidation-reduction process;carbon fixation;monooxygenase activity;ribulose-bisphosphate carboxylase activity;plastid; |
| HORVU5Hr1G069040 | -8,525337932 | Tuliposide A-converting enzyme 1, chloroplastic | endoplasmic reticulum to Golgi vesicle-mediated transport;catalytic activity, acting on a protein;proteolysis;intracellular protein transport;2-hydroxyisoflavanone dehydratase activity;COPII vesicle coat;hydrolase activity; |
| HORVU5Hr1G063660 | -8,915337187 | Low molecular mass early light-inducible protein HV90, chloroplastic | organelle envelope;chloroplast;integral component of membrane;thylakoid membrane;plastid part;organelle membrane;organelle subcompartment; |
| HORVU1Hr1G000350 | -8,938036819 | Jasmonate induced protein | integral component of membrane; |
